# Prevalence and Epidemiology of Non-O157 Escherichia coli Serogroups O26, O103, O111, and O145 and Shiga Toxin Gene Carriage in Scottish Cattle, 2014–2015

**DOI:** 10.1128/AEM.03142-20

**Published:** 2021-04-27

**Authors:** Deborah V. Hoyle, Marianne Keith, Helen Williamson, Kareen Macleod, Heather Mathie, Ian Handel, Carol Currie, Anne Holmes, Lesley Allison, Rebecca McLean, Rebecca Callaby, Thibaud Porphyre, Sue C. Tongue, Madeleine K. Henry, Judith Evans, George J. Gunn, David L. Gally, Nuno Silva, Margo E. Chase-Topping

**Affiliations:** a The Roslin Institute and Royal (Dick) School of Veterinary Studies, University of Edinburgh, Easter Bush, UK; b Moredun Research Institute, Edinburgh, UK; c Scottish *E. coli* O157/STEC Reference Laboratory, Department of Laboratory Medicine, Royal Infirmary of Edinburgh, Edinburgh, UK; d The Pirbright Research Institute, Pirbright, Woking, UK; e Laboratoire de Biométrie et Biologie Evolutive, Université de Lyon, Villeurbanne, France; f Epidemiology Research Unit, Scotland’s Rural College (SRUC), An Lòchran, Inverness Campus, Inverness, UK; INRS—Institut Armand-Frappier

**Keywords:** Shiga toxin-producing *Escherichia coli*, non-O157, cattle, epidemiology

## Abstract

Cattle are a reservoir for Shiga toxin-producing Escherichia coli (STEC), zoonotic pathogens that cause serious clinical disease. Scotland has a higher incidence of STEC infection in the human population than the European average. The aim of this study was to investigate the prevalence and epidemiology of non-O157 serogroups O26, O103, O111, and O145 and Shiga toxin gene carriage in Scottish cattle. Fecal samples (*n* = 2783) were collected from 110 herds in 2014 and 2015 and screened by real-time PCR. Herd-level prevalence (95% confidence interval [CI]) for O103, O26, and O145 was estimated as 0.71 (0.62, 0.79), 0.43 (0.34, 0.52), and 0.23 (0.16, 0.32), respectively. Only two herds were positive for O111. Shiga toxin prevalence was high in both herds and pats, particularly for *stx*_2_ (herd level: 0.99; 95% CI: 0.94, 1.0). O26 bacterial strains were isolated from 36 herds on culture. Fifteen herds yielded O26 *stx*-positive isolates that additionally harbored the intimin gene; six of these herds shed highly pathogenic *stx*_2_-positive strains. Multiple serogroups were detected in herds and pats, with only 25 herds negative for all serogroups. Despite overlap in detection, regional and seasonal effects were observed. Higher herd prevalence for O26, O103, and *stx*_1_ occurred in the South West, and this region was significant for *stx*_2_ at the pat level (*P = *0.015). Significant seasonal variation was observed for O145 prevalence, with the highest prevalence in autumn (*P = *0.032). Negative herds were associated with Central Scotland and winter. Herds positive for all serogroups were associated with autumn and larger herd size and were not housed at sampling.

**IMPORTANCE** Cattle are reservoirs for Shiga toxin-producing Escherichia coli (STEC), bacteria shed in animal feces. Humans are infected through consumption of contaminated food or water and by direct contact, resulting in serious disease and kidney failure in the most vulnerable. The contribution of non-O157 serogroups to STEC illness was underestimated for many years due to the lack of specific tests. Recently, non-O157 human cases have increased, with O26 STEC of particular note. It is therefore vital to investigate the level and composition of non-O157 in the cattle reservoir and to compare them historically and by the clinical situation. In this study, we found cattle prevalence high for toxin, as well as for O103 and O26 serogroups. Pathogenic O26 STEC were isolated from 14% of study herds, with toxin subtypes similar to those seen in Scottish clinical cases. This study highlights the current risk to public health from non-O157 STEC in Scottish cattle.

## Abstract (precis)

Cattle are reservoirs for Shiga toxin Escherichia coli (STEC), bacteria shed in animal feces. Humans are infected through consumption of contaminated food or water and by direct contact, causing serious disease and kidney failure in the most vulnerable.

## INTRODUCTION

Shiga toxin-producing Escherichia coli (STEC) strains are zoonotic bacteria that cause a spectrum of disease in humans, from asymptomatic carriage or mild diarrhea through to hemorrhagic colitis and hemolytic uremic syndrome (HUS) (1). The bacteria produce several virulence factors, of which Shiga toxin (Stx) is the predominant causative factor responsible for severe pathology. Stx is expressed by lysogenic bacteriophages incorporated into the bacterial genome (2). There are two known types of Stx, encoded by the *stx*_1_ and *stx*_2_ genes; variant subtypes of these genes occur (3), with the subtypes *stx*_2a_, *stx*_2c_, and *stx*_2d_ more commonly associated with serious disease (1, 4). A subset of STEC strains, designated enterohemorrhagic E. coli (EHEC), additionally carry the *eae* gene for the virulence factor intimin, which mediates attachment to the intestinal mucosa and increases infection severity (1, 5).

In 1982, E. coli O157 was the first STEC serogroup identified as the causal pathogen in human cases of severe hemorrhagic colitis and HUS (6). The majority of O157 STEC strains differ from commensal and other pathogenic E. coli bacteria in their inability to ferment sorbitol, a characteristic exploited by selective culture media, enabling O157 STEC to be distinguished from other serogroups (7). Public health surveillance programs based on such methodologies were developed around the world from the late 1980s in order to aid diagnosis and track the epidemiological spread of O157 (1, 8).

There are many non-O157 serogroups that also carry *stx* genes and cause severe human disease (9). Due to the lack of specific diagnostic tests, for many years the contribution of these non-O157 STEC to clinical disease has been substantially underestimated (10). However, recent progress in the development of culture-independent molecular assays that detect *stx* and other virulence genes has markedly increased the number of infections attributed to non-O157 STEC worldwide (11). Non-O157 STEC strains are now frequently identified as the primary pathogen in both sporadic and clinical outbreaks of severe disease, including HUS and death (4, 12). Consequently, a new classification system has recently been proposed, the JEMRA scheme (13), based upon virulence gene composition. Under this scheme, STEC strains carrying the *stx*_2a_ gene (with or without *stx*_1a_) together with *eae* are classified as JEMRA level 1, the most pathogenic tier, capable of inducing bloody diarrhea and HUS.

While in many countries O157 STEC remains the single most commonly reported serogroup in clinical cases, the proportion of STEC illness attributed to combined non-O157 serogroups now exceeds that attributed to O157. The predominance of non-O157 STEC has become evident over the past decade, particularly in Ireland (14), the wider European Union/European Economic Area region (15), and the United States (16–18). This is due in part to the change in methodology of molecular-based assays, as well as to mandatory requirements to report non-O157 STEC cases in many countries. O26 is the second most frequently reported STEC serogroup, and in some countries, including Ireland, France, Italy, and Denmark, clinical O26 cases currently surpass those due to O157 (19). Non-O157 infections appear to be of particular clinical significance in pediatric patients, in whom disease severity is similar to that of O157 (20, 21). Recent outbreaks of highly pathogenic *stx*_2_^+^ *eae*^+^ O26:H11 serotype strains have caused multiple HUS cases in young children, notably in France, Italy, and Romania (22–25). O26:H11 strains now account for a greater proportion of HUS cases than do O157 STEC strains in the European Union (12) and are of emerging importance.

Within the United Kingdom, Scotland has a particularly high incidence of STEC infection, and in 2019 the reported rate for all STEC was 4.7 per 100,000 population, approximately double the European Union average (12, 26). The proportion of non-O157 STEC cases has increased over the last decade in Scotland, accounting for approximately 40% of clinical reports since 2018 (26). In England, there is no standardized screening for non-O157 STEC; however, between 2013 and 2017, a regional survey reported that non-O157 serogroups comprised more than 80% of clinical isolates (27). Non-O157 STEC strains therefore represent an increasing public health concern.

Ruminants are the primary animal reservoir for STEC, predominantly farmed cattle and sheep (28), although recent reports of STEC isolation from wild animals, including deer and birds, suggest widespread dissemination of these pathogens across differing environmental niches (29–31). Generally, STEC bacteria do not cause significant morbidity or affect productivity in livestock and have therefore not been an economic priority for farm-level control. Management of transmission to humans is currently reliant on hygiene interventions by food business operators and public education on the risks associated with consumption of raw or undercooked meat and unpasteurized dairy products. Control within rural environments depends on hygiene measures such as hand-washing, together with an awareness of the risks posed by livestock contact and untreated water sources.

With the current rise in clinical cases attributed to non-O157 serogroups, livestock surveillance remains an important tool for measuring public health risk, as recommended in the Scottish Government Action Plan (32). Prevalence estimates provide an indication of the size of potential animal reservoirs, while epidemiological analyses aid the identification of geographic and husbandry factors that may be associated with livestock carriage. Between 2002 and 2004, a cross-sectional survey of beef cattle in Scotland estimated O26 herd prevalence to be equivalent to that of E. coli O157, at 23% (33). A new national survey was conducted in 2014–2015, the British E. coli O157 in Cattle Study (BECS) (34). BECS highlighted the stable epidemiological level of O157 STEC in Scottish cattle, with carriage observed in about one-fifth of sampled herds.

In this study, we aimed to assess current prevalence levels for O26, O103, O111, and O145 serogroups, together with *stx* gene carriage, in the Scottish cattle component of BECS using a PCR-based method (35). Determining cooccurrence of *stx* and target O groups is important for assessing the potential risk of new *stx* strain emergence, where receptive serogroup backgrounds and *stx* gene reservoirs coincide (36). We investigated possible risk factors associated with herd prevalence, such as region, season, and farm management. Finally, we isolated O26 strains from positive herds in order to assess the proportion shedding pathogenic *stx*-positive strains.

## RESULTS

### Overall pat- and herd-level prevalence, Scotland, 2014–2015.

A total of 110 herds and 2,783 pat samples (median of 23 pats per herd, range 1 to 75) were tested by real-time PCR for the *stx*_1_, *stx*_2_, O26 *wzx*, O103 *wzx*, O111 *wbdL*, and O145 *wzy* genes (Table 1). Shiga toxin gene carriage was high in the study population; over the 110 sampled herds, all but one were positive for *stx*_2_ and 91 were positive for *stx*_1_. Similarly, over the 2,783 pat samples, 70.2% and 40.2% were positive for *stx*_2_ and *stx*_1_, respectively. The most frequently observed serogroup in the study population was O103, followed by O26 and then O145. In contrast, O111 was rare, detected in less than 0.2% of pats, in only two herds.

**TABLE 1:** The number of study herds and pats testing PCR positive for Shiga toxin genes and the four non-O157 serogroups O26, O103, O111, and O145

Target	Herd (*n* = 110)		Pat (*n* = 2,783)	
	*n*	%	*n*	%
*stx*_1_	91	82.7	1,118	40.2
*stx_2_*	109	99.1	1,955	70.2
O26	47	42.7	420	15.1
O103	78	70.9	1,102	39.6
O111	2	1.8	4	0.2
O145	25	22.7	152	5.5

**FIG 1:**
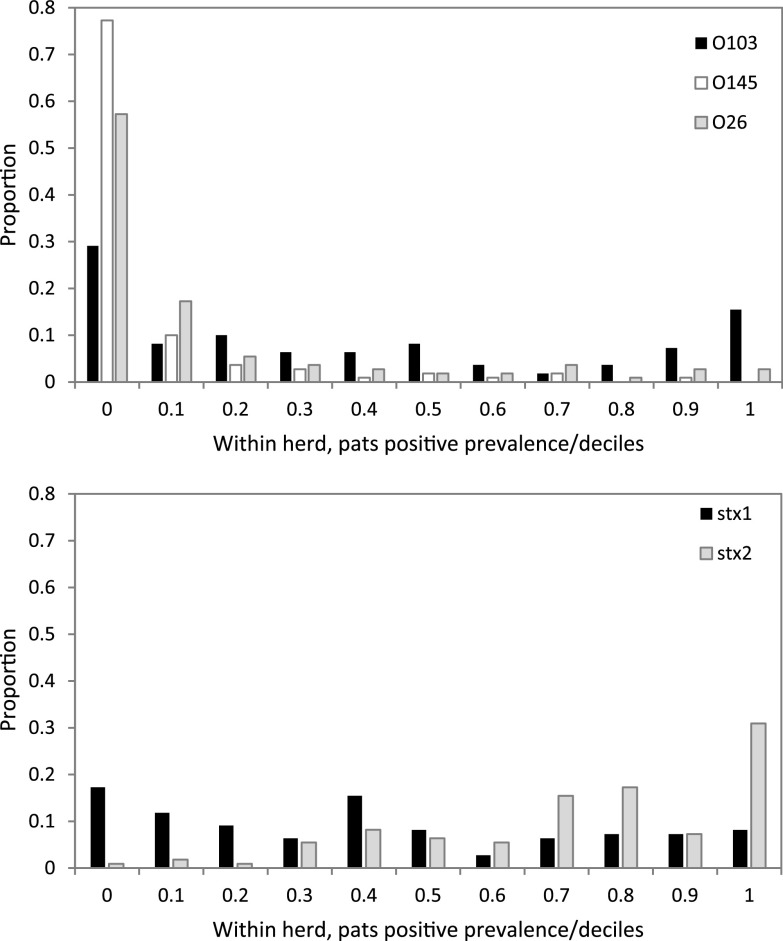
Within-herd prevalence distribution for O26, O103, and O145 serogroups, and *stx*_1_ and *stx*_2_ gene targets, as proportion of pats (*n* = 2783) positive per herd (*n* = 110), in decile categories up to 1.0 (*x* axis) versus proportion of herds (*y* axis).

We examined how within-herd prevalence for O26, O103, O145, and *stx* genes may vary between herds (Fig. 1). For O26 and O145, the distribution was highly skewed right, with more than 80% and 90% of herds, respectively, showing a within-herd prevalence of <20%. The within-herd prevalence for O103 showed a bimodal distribution, with 47% of herds having <20% positive pats but with 23% of herds showing over 80% positive pats (median 25%, range 0 to 100%). The distribution for *stx*_2_ was skewed left, and 38% of herds had a within-herd prevalence of over 80%. In contrast, *stx*_1_ showed a more even distribution in pat prevalence across herds (median 36%, range 0 to 100%).

**FIG 2:**
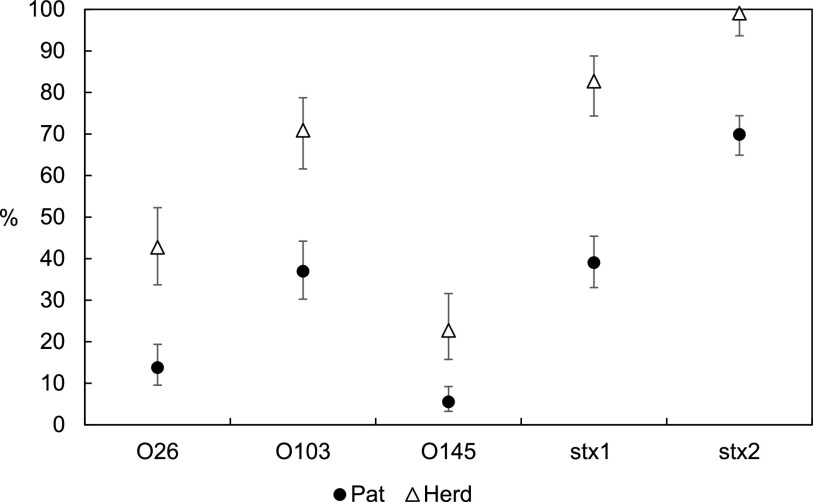
Prevalence estimates for herds (*n* = 110) and pats (*n* = 2,783) testing PCR positive for O26, O103, and O145 serogroups and *stx* genes, Scotland, 2014–2015. Error bars denote 95% CI.

A generalized linear mixed model (GLMM) was used to estimate herd- and pat-level prevalence values for Scotland (Fig. 2 and Tables S3 and S7). The mean herd-level prevalence estimates (95% CI) obtained were 70.9% (61.6, 78.7) for serogroup O103, 42.7% (33.7, 52.3) for O26, and 22.7% (15.8, 31.6) for O145. It was not possible to generate an estimate for the O111 serogroup due to low frequency. *stx* gene carriage was ubiquitous throughout Scotland, estimated at the herd level as 82.7% (74.4, 88.8) for *stx*_1_ and 99.1% (93.7, 99.9) for *stx*_2_.

### Seasonal and geographical distributions.

Seasonal effects on gene carriage were examined across the four seasons of the year. The highest herd prevalence occurred during the autumn for O26, O103, and O145 and was lowest during the winter months for O103 and O145 and during spring for O26 (Table S3). For *stx*_1_, the highest prevalence was in the spring. Season was analyzed within the GLMM across all serogroups; although the estimates for herd prevalence were highest in the autumn for O26, O145, and O103, this was only statistically significant for O145 (overall *P* value of 0.004; pairwise autumn to winter *P* value of 0.032).

**FIG 3:**
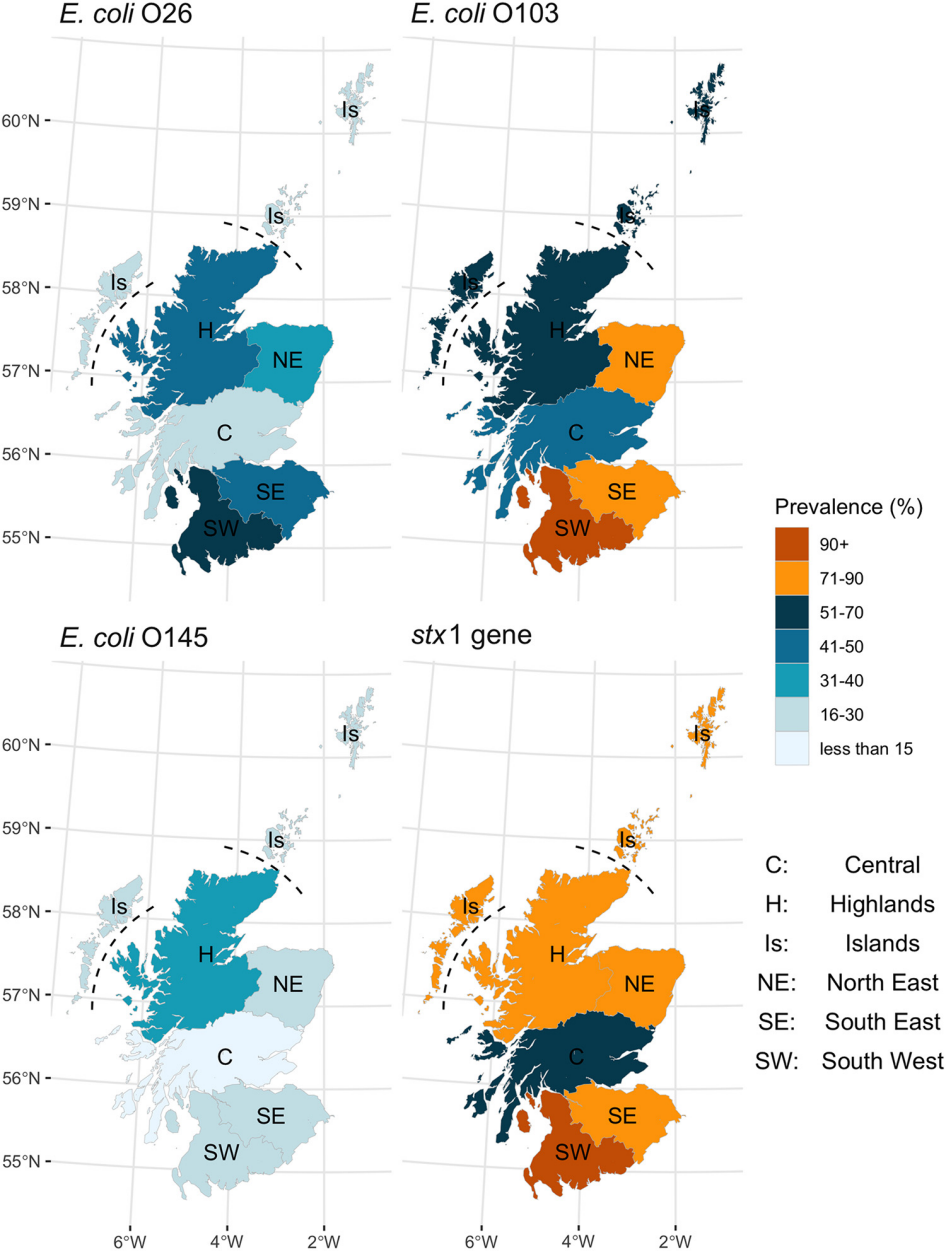
Observed herd prevalence by Animal Health District region for E. coli O26, O103, O145, and *stx*_1_ by real-time PCR, Scotland, 2014–2015.

The prevalence of positive herds by animal health district (AHD) was examined to investigate possible spatial variation across Scotland. The herd-level prevalence within the study population was highest in the South West of Scotland for O26, O103, and *stx*_1_ and was lowest in the Central areas for O103 and *stx*_1_ and in the Central and Islands areas for O26 (Fig. 3). There was no apparent regional distribution in O145-positive herds, although this was lowest in the Central AHD. Regional herd- and pat-level estimates were calculated within the GLMM (Tables S3 and S7). The highest herd prevalence for O26 and O103 was estimated for the South West, and the lowest was estimated in the Central and Islands areas. All regions showed a high prevalence at the herd level for *stx*_1_, highest in the South West and lowest in Central. It was not possible to examine herd-level spatial distribution for *stx*_2_, since most herds tested positive (109/110); however, there was a statistically significant difference in *stx*_2_ pat-level prevalence between regions (*P = *0.015). The highest *stx*_2_ pat prevalence was in the South West, 81.1% (95% CI: 72.3, 87.6), and the lowest was in the Islands, 56.3% (95% CI: 41.7, 69.9) (pairwise comparison, *P = *0.03). The two positive O111 herds were located in the South West and South East, while the single *stx*_2_-negative herd was present in the Islands.

### Multiple serogroups and *stx* within herds and pats.

The majority of herds showed a positive result for at least one of the target serogroups, with only 21.8% of herds (*n* = 25) negative for all serogroups tested, 34.5% (*n* = 39) positive for a single serogroup, and 41.8% (*n* = 46) positive for two or more serogroups. The combination of O26 and O103 was the most commonly detected pattern at the herd level. Tetrachoric correlation analysis showed the strongest correlation between O103 and O145 serogroups (*r*_tet_ = 0.99), with all O145-positive herds also testing O103 positive. A moderate association was also observed between O26 and O145 (*r*_tet_ = 0.64) and for O26 and O103 (*r*_tet_ = 0.57) serogroups. Both O111-positive herds were also O26 and O103 positive, with one additionally O145 positive. Regionally, Central and the Islands had the greatest number of herds negative for the four non-O157 serogroups (37.5%) investigated, while there were no herds in the South West negative to all.

**FIG 4:**
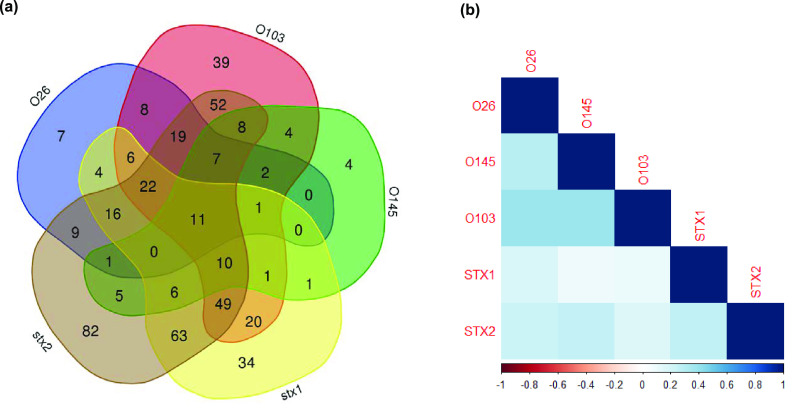
Positive pat PCR target combinations observed across all herds (*n* = 110) for *n* = 491 sample events depicting (a) Venn diagram showing the frequency of all possible combinations for O26, O103, O145, *stx*_1_, and *stx*_2_ and (b) tetrachoric correlation results between paired targets.

**FIG 5:**
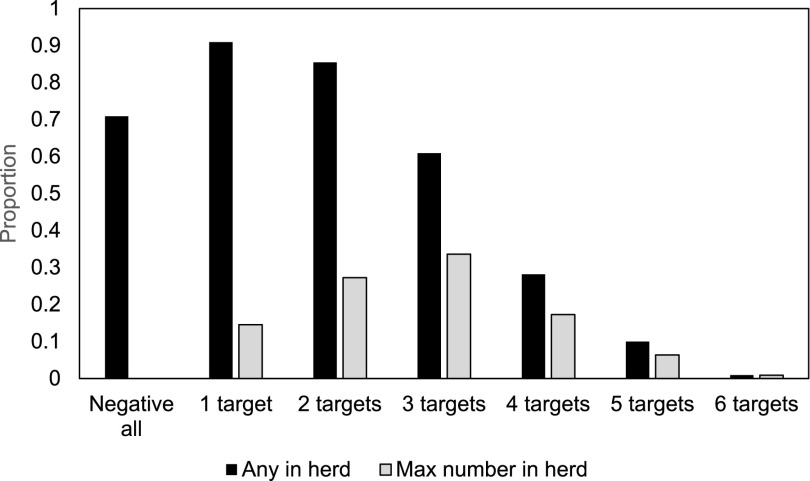
The proportion of herds (*n* = 110) with at least one sample event in herd for the number of targets shown (“Any in herd”), and the maximum number of targets per sample observed in a herd (“Max number in herd”).

To examine within-sample combinations for a positive PCR status between the four serogroups and *stx*, a single index event per herd for each combination of the six genes tested was recorded. Further occurrences of a combination were excluded to prevent herds with large numbers of pats from dominating the analysis. A total of 31 possible PCR gene target combinations were observed across 491 combination events, with a median per herd of four different combinations (range, 1 to 12) (Fig. 4a). For all positive pats, 33.8% were positive to both 1 and 2 targets, 21.8% were positive to 3, 8.2% to 4, 2.2% to 5, and only 0.2% to all 6 targets. The most common pattern seen was *stx*_2_ (16.7% of events), followed by *stx*_1_ *stx*_2_ (12.8%) and O103 *stx*_2_ (10.6%). Tetrachoric correlation analysis showed a low positive correlation among all the serogroups and *stx* (Fig. 4b). The highest association was observed between O103 and either O145 (*r*_tet_ = 0.39) or O26 (*r*_tet_ = 0.38). For *stx*_2_, the highest correlation was for O145 (*r*_tet_ = 0.25) and O26 (*r*_tet_ = 0.23). Due to low prevalence, correlation was not performed for O111. Thirty-two herds (29.1%) gave a positive result to at least one gene target for every pat sample tested, and 58.2% (*n* = 64) of herds had samples positive to three or more targets (Fig. 5). There were no herds for which every single pat tested was negative to all six of the PCR targets.

### Comparison with historical farm data.

There were 80 herds in the current study that had also been previously screened for non-O157 serogroups in the 2002 to 2004 cross-sectional survey (33). No significant association was observed between previous historical and current herd status (Table 2) for O26 (Pearson’s χ^2^ = 0.42, *P = *0.52), O103 (Pearson’s χ^2^ = 0.007, *P = *0.93), or O145 (Fisher’s exact test, *P = *1.0).

**TABLE 2:** Comparison of current and historical herd serogroup status for all herds (*n* = 80) tested in both the 2002 to 2004 and 2014–2015 surveys

Serogroup	Herd comparison at both time points		
	Positive both	Negative both	Changed status
O26	10	35	35
O103	12	20	48
O145	1	18	61

### Herd risk analysis.

Nonmetric multidimensional scaling (NMS) was used to identify epidemiological survey variables associated with the presence of the non-O157 serogroups (O26, O145, and O103) and STEC O157. The final NMS solution was two-dimensional (2D) and explained 80.0% (cumulative *r*^2^ = 0.800, axis 1 *r*^2^ = 0.587 and axis 2 *r*^2^ = 0.216) of the variation in the presence of E. coli serogroups in herds. More variation than expected occurred by chance (Monte Carlo test *P = *0.004). Final stress for the two-dimensional solution was 4.09 (final stress <5 suggests an excellent representation) (37) and final instability was 0, with 51 iterations. Number of iterations is the number of steps that NMS performed to find the final solution (38).

**FIG 6:**
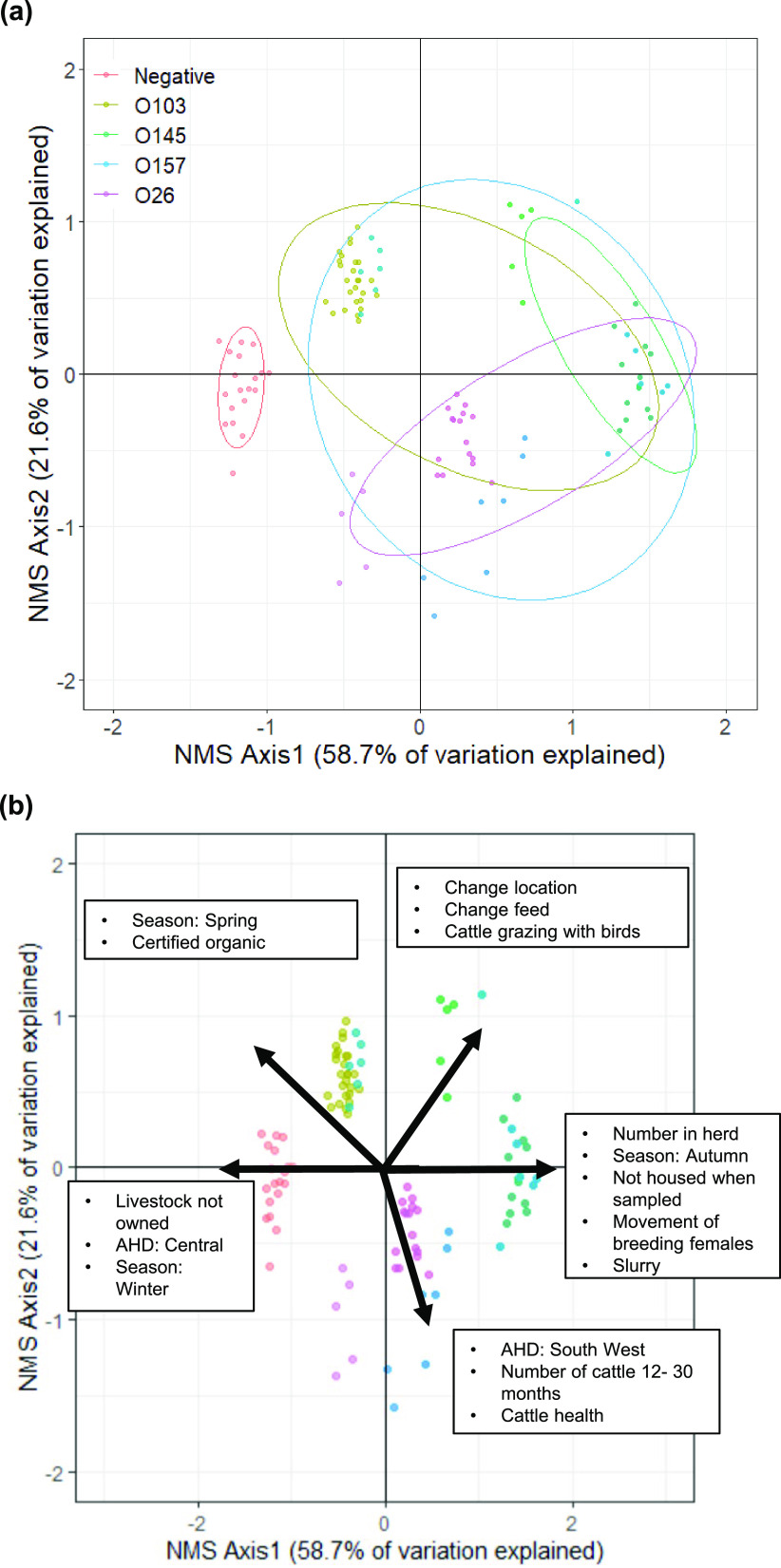
Nonmetric multidimensional scaling (NMS) ordination of herd-level serogroup results. (a) Scatterplot and 75% confidence ellipses round the herds in space illustrating the extent of overlap between herds with O103, O26, O145, and STEC O157. (b) All epidemiological variables associated with NMS axes 1 and 2 are shown as vectors radiating from the centroid; the direction indicates positive or negative association on that axis. AHD denotes Animal Health District.

The results are shown using 2D ordination graphs of the distance between sample units, which approximate dissimilarity in the serogroup presence in herds (Fig. 6). The *x* axis explains most of the variation in the data (58.7%) and represents herds with no serogroup identified (left) and herds where all serogroups were identified (right). Confidence ellipses (75%) highlight herds with each serogroup (Fig. 6a). There is considerable overlap in ellipses, indicating that many herds were positive for more than one serogroup; however, there was a trend for herds with O26 serogroups in the lower quadrant and herds with O103 and O145 in the upper quadrant. STEC O157 was associated with herds in all quadrants. Factors associated with each axis are shown as vectors (Fig. 6b); the direction indicates positive or negative correlation (not strength) along the axis. Negative herds were associated with the Central AHD, winter, and herds with livestock not owned by the farm. Herds with all serogroups identified were associated with greater numbers of cattle in the herd, autumn, not being housed at the time of sampling, slurry spreading, and movement of breeding females into the herd. Herds with both O103 and O145 were associated with potential biosecurity-related variables, such as bringing cattle into the herd, change in location or of feed, and cattle grazing with birds. Herds with O26 were associated with larger numbers of cattle in the herd aged 12 to 30 months, the South West (AHD), and reports of cattle health issues within the last 2 weeks.

### Isolation and identification of O26 strains from PCR-positive herds.

We attempted isolation of O26 strains from all herds that were O26 PCR positive, using an immunomagnetic separation (IMS) technique. IMS was performed on a maximum of eight pat samples per herd, selecting those with the lowest recorded PCR threshold cycle (*C_t_*) in order to maximize yield. Strain isolation was not attempted from every PCR-positive pat in a given herd, since this was beyond the scope of the current study.

O26 strains were isolated from 36 of 47 herds that were O26 PCR positive. The IMS confirmation rate for PCR-positive herd status was therefore 77%, and a total of 196 O26 isolates were obtained from 64 pat samples. For herds yielding a positive O26 isolate, the mean number of PCR-positive pats in the herd was 10, compared to 4 positive pats per herd for those that did not yield an O26 isolate. Furthermore, herds yielding an O26 isolate had a significantly lower minimum sample *C_t_* value than that of herds that failed to generate an O26 isolate (median *C_t_* value of 29.5 versus 33.2, Mann-Whitney U test, *P = *0.004).

Of the 36 herds that generated O26 isolates, 21 herds yielded O26 *stx*-negative isolates only and 15 herds yielded O26 *stx*-positive isolates (*n* = 67). Real-time PCR and Illumina sequencing confirmed that of the O26 *stx*-positive herds, nine generated O26 *stx*_1a_-only isolates, three gave dual-positive *stx*_2a_-*stx*_1a_ isolates, one gave dual-positive *stx*_2d_-*stx*_1a_ isolates, and two yielded O26 stx_2a_-only isolates. All the O26 *stx*-positive isolates were additionally positive for the intimin gene, *eae*. By region, the proportion of all herds yielding O26 strains that gave an *stx*-positive isolate was greater in the Highlands (0.57) and South West (0.5) than in all other regions (0.3), although this difference was not statistically significant.

Systematic IMS for isolation of O103 and O145 strains from positive farms was beyond the scope of this study, due to operational constraints. However, O103 strains (22 isolates) were isolated from samples in seven herds that were undergoing O26 IMS. All O103 isolates were *stx* negative, and in three herds, both O103 and O26 were isolated jointly from the same pats. In addition to the targeted O26 strains, *stx*-positive strains of other O serogroups were isolated incidentally from samples in six O26-shedding herds. These strains were identified as *stx*_1a_-positive O84, O98, O149, O177, and O182, as well as O136 (*stx*_2a_) and O150 (*stx*_2a_-*stx*_1a_). Coisolation of strains with O26 from the same individual pat sample occurred in five of these herds, including individual pats from two herds for which three serogroups were obtained: O26 plus O103, with either O155, O177, or O182.

## DISCUSSION

### Prevalence of *stx* and O serogroups.

In Scotland, the frequency of observed Shiga toxin gene carriage in cattle was high, with 99% and 83% of herds and 70% and 40% of pats being *stx*_2_ and *stx*_1_ positive, respectively. Shiga toxin gene carriage was therefore ubiquitous in this study population. To our knowledge, the prevalence of *stx* has not been previously estimated for Scottish cattle; the data highlight the widespread distribution of *stx* gene carriage in this reservoir. Since we used a culture-independent method, the observed prevalence encompasses *stx* harbored both by STEC organisms, i.e., with *stx* virulence genes incorporated into the bacterial genome, and in free Stx-encoding bacteriophage. Such high carriage by cattle has been reported in several countries, including the United States, Canada, Europe, and Australia (39–44). Across all these studies, the prevalence of *stx*_2_ is greater than that of *stx*_1_, similar to our own observations. Comparing exact prevalence values between studies is not always pertinent, due to differences in screening methodology and sample population demographics; however, despite this, the concurrence in high *stx* prevalence observed in this survey is notable.

The most common serogroup detected at both herd and pat levels was O103, followed by O26 and then O145, with O111 detected in only two herds. This order of frequency differs from that of the 2002 to 2004 survey (33), in which O26 and O103 were of similar estimated herd prevalence (22 to 23%). Both surveys agreed on the ranking of O145 and O111, with O111 not previously detected. We estimated a higher PCR-based prevalence for all three serogroups at both herd and pat level, probably due to the increased sensitivity of real-time PCR compared to that of the previous culture-based methodology. Molecular techniques are now routinely employed for STEC diagnostics in both clinical and food safety environments (1) and are particularly useful for identifying non-O157 STEC infection, since these serogroups are difficult to distinguish on culture from commensal E. coli. We did confirm O26 herd status by strain isolation, using a similar IMS methodology to that of the 2002 to 2004 survey but with a change in culture medium to CHROMagar STEC, an agar that supports growth of a wider range of STEC serogroups (45). O26 shedding was confirmed in 77% of the PCR-positive herds, i.e., 33% of the overall study population. This is an increase compared to the proportion of herds yielding O26 strains previously and may be due to a genuine increase in O26 herd carriage within Scotland or the result of differences in methodology, such as the change in selective agar.

The high prevalence of O103 positivity was unexpected but is corroborated by other studies, with O103 found to be a predominant non-O157 serogroup in dairy cattle in New Zealand (46) and Ireland (47) and in beef cattle in the United States (48, 49), Canada (50), and Australia (44). Despite high serogroup prevalence, recovered O103 isolates from cattle appear to carry *stx* genes relatively infrequently (44, 46, 47, 49, 51), as also observed in the previous Scottish survey (33). O103 would therefore appear to be of lower public health concern.

We report an overall serogroup prevalence and not STEC-specific O group prevalence. Stx-encoding bacteriophage are mobile and may exist freely, while lysogeny enables incorporation into receptive E. coli backgrounds, allowing Shiga toxin expression with subsequent pathogenic effect (35, 52). Where reservoirs of Stx-encoding bacteriophage coincide with receptive serogroup backgrounds, there exists the potential for evolution of emergent STEC strains. In particular, O26 *stx* status is known to be dynamic, with loss and acquisition of *stx* phage demonstrated *in vitro* and *in vivo* (53–56). Individual pat samples from two herds in our study yielded both *stx*-negative and -positive O26 isolates, suggesting that the source animal was colonized either by two differing O26 strains or by the same strain from which *stx* genes had been lost or acquired within the gut or during laboratory culture. Without screening all available pats and a greater number of colony picks per sample, we cannot definitively state that any given herd was O26 *stx* negative.

### Multiple serogroup detection.

There was a positive correlation observed between the detection of multiple serogroups at both herd and pat levels. All herds that were O145 positive formed a subset of O103-positive herds, and the majority of O26-positive herds were also O103 positive. Coshedding of O26 and O103 within management groups had been previously observed in the 2002 to 2004 study (33). The increased sensitivity of PCR here enabled demonstration of cocarriage within individual samples, confirmed in a small subset by isolation of both O26 and O103 from individual pats. Other STEC serogroups were also isolated incidentally during IMS, with two herds each yielding three different serogroups from individual pats. Cocarriage of non-O157 serogroups in individual cattle is reported elsewhere (57, 58), while a significant positive association between O103 with O26 or O145 carriage has been observed in individual calves (51). Serogroup association is likely a reflection of common environmental and husbandry factors independently favoring carriage of multiple E. coli serogroups, although the possibility of serogroup facilitation within niches cannot be discounted.

### Comparison with human data in Scotland.

Data from an equivalent reporting period, 2014–2015, show that O26 was the commonest non-O157 STEC isolated from clinical submissions to the Scottish E. coli O157/STEC Reference Laboratory (SERL), comprising 25.4% of total cases, followed by O145 (10.7%) and O103 (6.0%) (personal communication, Lesley Allison). In the current cattle survey, while the proportion of herds that were O103 PCR positive was much higher than that of herds that were O26 positive, based on evidence from the 2002 to 2004 survey (33), the majority of these O103 herds were anticipated to be *stx* negative. These would be of limited risk to public health and therefore presumably less frequently identified in clinical samples. In contrast, O26 *stx*-positive strains were isolated from 11.2% of all sampled herds in 2002 to 2004 and from 13.6% of the herds in our current survey. Although the survey was not designed to be fully representative of the entire Scottish cattle population (59), we suggest that the cattle reservoir shedding O26 STEC in Scotland is substantial. This is reflected by the predominance of the O26 serogroup in non-O157 STEC clinical submissions to the SERL.

Further evidence that cattle are implicated as a reservoir for human O26 STEC infection is given by the *stx* subtype carried by isolates. Prior to 2010, all clinical O26 strains submitted to SERL harbored either *stx*_1a_ or the combination *stx*_2a_-*stx*_1a_ gene profiles; however, in 2010 the first clinical O26 *stx*_2a_-only strains were isolated (60). Since then, while the majority of clinical O26 strains continue to be either *stx*_1a_- or *stx*_1a_-*stx*_2a_-positive, a minority of O26 *stx*_2a_-only positive cases also occur. These data agree with the *stx* strain distribution observed within the two Scottish cattle surveys, in which all O26 STEC strains isolated between 2002 and 2004 were either *stx*_1a_- or dual *stx*_1a_-*stx*_2a_-positive, with no herds shedding *stx*_2a_-only strains. In contrast, despite the smaller survey sample size, in this study we identified two cattle herds shedding *stx*_2a_-only positive O26 strains. Overall, the distribution of *stx* subtypes in the O26 STEC shedding herds was very similar to that observed in clinical strains across the same time period (60). Isolates from 5 out of the 15 O26 STEC shedding herds met the criteria for JEMRA level 1 (13), while the remainder of the *stx*-positive herds yielded strains equivalent to JEMRA level 4. All the identified O26 STEC herds therefore shed bacteria of substantial public health risk.

### Seasonal, spatial, and risk factor analysis.

In general, prevalence was higher in the autumn for both O26 and O103; this was highly significant for O145. Ordination analysis indicated that herds positive for all serogroups were more associated with autumn, in cattle not housed at time of sampling, while those negative for all serogroups were associated with winter. Similar seasonal prevalence of non-O157 is observed elsewhere, including North America (49, 50, 61) and Ireland (62), with significantly lower prevalence observed in cattle in colder winter conditions and the highest prevalence observed during summer and early autumn. The clinical picture also reflects these data, with higher summer-to-autumn incidence in human non-O157 case reports locally in Scotland (26), in wider Europe (12, 63), and in the United States (18), suggesting that human acquisition is directly influenced by seasonal abundance in the animal reservoir. Seasonal variability could also be influenced by climate factors, such as rainfall and ground temperature, together with management practices, including whether animals are housed or grazed.

The highest herd-level prevalence for O26, O103, and *stx*_1_ was observed in the South West of Scotland, with prevalence lowest in the Central and Island regions; within the ordination analysis, this effect was greatest for the O26 serogroup. *stx*_2_ pat-level prevalence showed a statistically significant regional effect, with higher positivity in the South West than in the Islands. The density and distribution of cattle farms by management type are not uniform throughout Scotland, due to geographical and climatic factors. Dairy herds are concentrated particularly in the South West, with this region accounting for 78% of such farms (64). This is reflected in the BECS study demographic, with 10/14 of the dairy herds sampled in this survey based within the South West AHD, comprising 43% of the herds tested in the region (34). In contrast, no dairy farms were sampled in the Islands, Highlands, or North East AHDs. Knowledge of regional health risks is important for local disease control and could aid the targeted introduction of public health measures, such as increased herd biosecurity, public health education, and potential vaccination (65).

The role of dairy cattle in zoonotic transmission of STEC has gained recent prominence, with several O26 STEC outbreaks in Europe linked to consumption of unpasteurized dairy products (23–25). In New Zealand, a recent survey found that herds positive for O26, O103, and O145 mapped to regions with a greater proportion of dairy farms (58). While STEC strains are not generally recognized as primary cattle pathogens, *stx*-positive serogroups, particularly O26, are frequently isolated from both healthy and diarrheic calves (28, 66) and may therefore be more common in environments with high calf production and stocking densities, which are typical in the dairy sector. Local area cattle density also appears to be of importance, with studies in both Germany (67) and Ireland (68, 69) demonstrating a significant association between human clinical non-O157 STEC case reports and regions with high local cattle densities.

Both climate and topography influence farm management type in Scotland, with land of poor productivity and grazing quality typically in colder upland regions more suited to extensive beef and sheep farming. Regionally, the South West tends to have a milder climate than that of other areas of Scotland and has relatively high annual rainfall (70). Climate, geography, and farm management type are therefore intrinsically linked, which confounds our ability to distinguish which factors may influence pathogen prevalence.

Additional farm variables associated with serogroup positivity included biosecurity-type factors such as movement of stock and feed for both O103 and O145. For O26, the number of cattle aged 12 to 30 months and herds reporting health issues were relevant variables. Cattle not housed at the time of sampling, slurry spreading, overall herd size, and the introduction of breeding females onto farms were associated with herds testing positive for all serogroups examined. Farm movements increase opportunities for both introduction and dispersal of bacteria, while high local density on a farm provides favorable conditions for animal-to-animal transmission (58). For the Scottish data set within the BECS survey, the introduction of breeding females into a herd and the number of cattle aged 12 to 30 months were also identified as risk factors that increased the odds of a herd testing O157 positive (59). The agreement between both the O157 and the non-O157 studies is notable and further substantiates the relevance of such factors in herd colonization.

### Limitations.

Discrepancy between the detection of gene signal within samples and isolation of viable strains is well established within the STEC field (71, 72). Strain isolation confirmed 76.7% of the O26 PCR-positive farms, with those herds not confirmed having a significantly higher minimum *C_t_* value than that of herds yielding strains. This isolation rate compares favorably with that reported for clinical isolation (26) and other livestock studies (46, 47, 51, 71). A number of practical reasons may account for failed isolation, including the low number of colony picks screened per pat, repeated freeze-thaw before reculture compromising viability, low bacterial concentration, and IMS bead efficacy (73, 74).

The farm samples used in this study were collected for the BECS study (34), the primary aim of which was to provide an estimate of E. coli O157 prevalence in Scottish beef cattle, comparable to historical surveys (75, 76). The sampling protocol was therefore powered to look at overall country prevalence of E. coli O157, and the detection of seasonal and spatial differences was not taken into account. This is reflected in the lack of statistical significance despite some clear spatial and temporal effects being observed in this study. A similar result was found for E. coli O157 (59).

### Conclusion.

This study has demonstrated widespread *stx* gene prevalence in cattle throughout Scotland. O103 was the most common serogroup, followed by O26, and only 25 farms were negative for all four non-O157 serogroups. Despite limited statistical power, geographical and seasonal effects were observed, with autumn being the most likely season to detect all serogroups. Compared to historical data, the prevalence for all non-O157 serogroups examined was higher, mirroring the current pattern observed in Scottish human clinical cases. All O26 STEC strains isolated were *eae* positive, with highly pathogenic *stx*_2_-positive strains identified in six herds. These data highlight the importance of cattle as a non-O157 STEC reservoir of risk to public health in Scotland.

## MATERIALS AND METHODS

### Sample source, study population, and herd epidemiological data.

Samples from Scottish cattle were provided for this study by the British E. coli O157 Cattle Study (BECS) (34) as 2-ml preprepared 6-h fecal pat enrichment cultures in buffered peptone water (BPW), stored at a final concentration of 15% (vol/vol) glycerol and frozen at −80°C. Individual bovine fecal pat samples (*n* = 2783) were collected over a 13-month period between September 2014 and September 2015 from 110 cattle herds throughout Scotland. Herds were randomly selected from a population of farms included in two previous surveys carried out between 2002 and 2004 (76) and from 1998 to 2000 (75). Sample size was estimated using the previous reported prevalence of E. coli O157, which was of an order similar to that of both O26 and O103 prevalence in the 2002 to 2004 survey (33), to ensure similar confidence in the current prevalence estimates. The number of samples (fresh fecal pats) collected from each herd was determined by the number of cattle in the sampling group using a prescribed sampling schedule (76). Individual herd data were obtained by questionnaire administered at the time of sample collection. Variables collected included herd size, management, health status, and stock movements. A detailed description of the data collected is outlined by Henry et al. (34), and a copy of the questionnaire is available upon request from the corresponding author.

### Bacterial control strains.

The following bacterial strains, used as positive controls for validation of real-time PCRs, were gifted by the Scottish E. coli O157/Reference Laboratory (SERL; Lesley Allison): Paton’s positive (PP+; for *stx*_1_, *stx*_2_ genes) from the Scottish National Culture Collection, H45 (for the *stx*_2f_ gene) from a 2011 European Centre for Disease Control (ECDC) External Quality Assurance (EQA) ringtrial, and O26, O103, and O145 serogroup strains from a 2005 ECDC EQA ringtrial. E. coli DEC/18B (from Tom Besser, Washington State University, United States) was used as the O111 serogroup control strain, and E. coli strain K-12 MG1655 (77) was used as a negative control.

### DNA extraction.

Frozen fecal enrichments were thawed on ice, and whole DNA was extracted using InstaGene matrix (Bio-Rad Laboratories Ltd., Watford, UK) spiked with phocine herpesvirus (PhHv) glycoprotein B (gifted by Lesley Allison, SERL) as an internal control to verify successful extraction and rule out the presence of PCR inhibitors (78). Fifty microliters of fecal enrichment culture was washed in 950 μl of nuclease-free water (Qiagen, Crawley, UK) and cells were pelleted by centrifugation at 13,000 rpm for 3 min. InstaGene, 200 μl, was added to the cell pellet, vortexed, and incubated in a heat block for 60 min at 56°C. Samples were vortexed again and boiled at 100°C for 20 min to ensure pathogen killing. The resultant DNA extract was cooled, the matrix was pelleted by centrifugation at 13,000 rpm for 1 min, and the DNA extract was stored frozen at −80°C until required. A repeat extraction procedure was routinely performed on every 20th fecal enrichment as a biological repeat (BR). For each daily batch of test samples processed, a DNA extraction was performed on overnight cultures in Luria-Bertani broth (LB broth; Oxoid Ltd., Basingstoke, UK) of PP+, H45, and MG1655 E. coli strains as positive and negative controls.

### Serial dilution of positive-control strains spiked into a negative fecal enrichment background for determining PCR cutoff.

Negative background fecal samples were obtained from young calves (79) that were prescreened PCR negative for all the relevant gene targets. The fecal samples were preenriched similarly to the test fecal pat samples, as described above, and stored frozen in aliquots as 15% (vol/vol) glycerol cultures at −80°C.

Ten-fold serial dilutions of positive strain cultures were prepared in LB broth and spiked into the negative fecal enrichment culture to provide a biologically relevant control for each PCR target, subject to any similar inhibitors that may have been present in the test sample fecal enrichments. The spiked fecal enrichment dilution series was used to determine equivalent designated cutoffs for allocating a sample to either positive or negative PCR status across the four O serogroups under investigation. We spiked feces at the postenrichment stage. Spiking at the preenrichment stage may have resulted in uneven dilution curves and nonequivalent serogroup cutoffs, due to possible differential growth of the four serogroup control strains within the complex bacterial base of the fecal enrichment culture. The limit of detection for each PCR target therefore relates to CFU at the 6-h postenrichment stage.

To spike the negative fecal enrichments, a single colony of each positive-control strain was grown individually overnight in 5 ml of LB broth at 37°C in a static incubator. The overnight culture was subcultured 1:100 in LB broth and grown with shaking (200 rpm) at 37°C for 4 h. For each control strain, a 10-fold serial dilution of the 4 h subculture was prepared in LB broth and individual dilution points were then spiked into aliquots of the negative fecal enrichment culture. Single strain spiked dilution series (10^−1^ to 10^−6^) were prepared for all individual control strains by spiking the respective 4-h subculture dilution point 1:10 into the negative fecal enrichment. Additionally, a triplex spiked dilution series (10^−2^ to 10^−6^) was prepared with O26, O103, and O111 control strains, spiking at a 1:100 ratio into the negative fecal enrichment. The control strain bacterial concentration (in CFU/ml) present in the 4-h subculture was determined by droplet count (80), spotting 10-μl droplets of each dilution point of the series onto sorbitol MacConkey agar plates (Oxoid), incubating overnight at 37°C, and performing colony counts the following day.

DNA extractions were prepared from each spiked fecal dilution control series using InstaGene matrix, as described above. Replicate extractions were performed for each dilution point and pooled, and batch aliquots were prepared for use as technical replicates in respective PCR assays. Aliquots were stored frozen at −20°C until required.

### Real-time PCR screening for Shiga toxin genes and O serogroups.

Each sample DNA extract was tested in three separate real-time PCR assays, following ISO guidelines (ISO/TS 13136:2012) (35), with some modifications developed by the SERL (81); primer and probe sequences are as described in Table 3. The reactions were as follows: (i) Shiga toxin multiplex for *stx*_1_, *stx*_2_, *stx*_2f_ variant, and PhHv, (ii) O145 singleplex, and (iii) O serogroup triplex for O26, O103, and O111.

**TABLE 3:** Primer and probe sequences for Shiga toxin, triplex O serogroup, and O145 real-time PCRs

Target	Primer and probe sequence, 5′ to 3′		Fragment size (bp)	Reference
Shiga toxin multiplex
*stx*_1_	Forward	TTTGTYACTGTSACAGCWGAAGCYTTACG	131	35
Reverse	CCCCAGTTCARWGTRAGRTCMACRTC			
Probe	5′:-Yakima Yellow-CTGGATGATCTCAGTGGGCGTTCTTATGTAA-BHQ1			
*stx*_2_	Forward	TTTGTYACTGTSACAGCWGAAGCYTTACG	128	35
Reverse	CCCCAGTTCARWGTRAGRTCMACRTC			
Probe	5′-FAM-TCGTCAGGCACTGTCTGAAACTGCTCC-BHQ1			
*stx*_2f_	Forward	TTGTCACAGTGATAGCAGAAGCTCTG	124	81
Reverse	CAGTTCAGGGTAAGGTCAACATCC			
Probe	5′-FAM-CGCTGTCTGAGGCATCTCCGCTTTATAC-BHQ1			
PhHv	Forward	GGGCGAATCACAGATTGAATC	89	78
Reverse	GCGGTTCCAAACGTACCAA			
Probe	5′-CY5-TTTTTATGTGTCCGCCACCATCTGGATC-BBQ			
O serogroup triplex (O26, O103, O111)
O26 *wzx*	Forward	CGCGACGGCAGAGAAAATT	135	35
Reverse	AGCAGGCTTTTATATTCTCCAACTTT			
Probe	5′-FAM-CCCCGTTAAATCAATACTATTTCACGAGGTTGA-BHQ1			
O103 *wzx*	Forward	CAAGGTGATTACGAAAATGCATGT	99	35
Reverse	GAAAAAAGCACCCCCGTACTTAT			
Probe	5′-CY5-CATAGCCTGTTGTTTTAT-BBQ			
O111 *wbdL*	Forward	CGAGGCAACACATTATATAGTGCTTT	146	35
Reverse	TTTTTGAATAGTTATGAACATCTTGTTTAGC			
Probe	5′-HEX-TTGAATCTCCCAGATGATCAACATCGTGAA-BHQ1			
O145 singleplex
O145 *wzy*	Forward	ATATTGGGCTGCCACTGATGGGAT	310	83
Reverse	TATGGCGTACAATGCACCGCAAAC			
Probe	5′-Texas Red-AGCAGTGGTTCGCGCACAGCATGGT-BHQ2			

Real-time PCR was performed in a reaction volume of 20 μl, in 96-well plates (Bio-Rad), comprising 10 μl QuantiTect Multiplex PCR NoROX master mix (Qiagen), 1 μl of 20× primer/probe mix, 7 μl of DNase-free water (Qiagen), and 2 μl of sample or control DNA template. The final primer concentration was 0.4 μM for all primers, except for *stx*_2f_ and PhHv (0.2 μM); final hydrolysis probe concentrations were 0.1 μM for *stx*_1_, *stx*_2_, *stx*_2f_, and PhHv and 0.2 μM for O26, O103, O111, and O145 probes. The primer annealing temperature was 60°C for the Shiga toxin multiplex and O145 singleplex reactions and 55°C for the triplex serogroup reaction (O26, O103, and O111). PCR was performed using a CFX 96 machine (Bio-Rad), with the following cycling conditions: initial denaturation cycle of 95°C for 15 min, followed by 40 cycles of 95°C for 15 s and the appropriate annealing temperature for 60 s. Primers and dually labeled hydrolysis probes were supplied by Eurofins Genomics (Wolverhampton, UK) and Eurogentec (Seraing, Belgium).

All test and control samples were loaded as duplicate wells. Each reaction plate included water as a no-template control, DNA for the appropriate positive-control dilution series, and negative-control strain MG1655 DNA. The triple (O26, O103, and O111)-spiked control series was used for the O serogroup triplex reaction after demonstrating that serogroup-specific *C_t_* values of triple-spiked cultures did not differ significantly from those of individual serogroup cultures at the equivalent dilution. Additional controls included the daily batch DNA extracts from control cultures of PP+, H45, and MG1655 for the Shiga toxin reaction and the 10^−1^ dilution point for each single culture O26, O103, and O111 series in the triplex O serogroup reaction.

### PCR data analysis.

CFX Manager software version 3.1 (Bio-Rad) was used to capture data. Identical fluorophore thresholds (relative fluorescence units) were set and applied to all plates for a given PCR, *stx*_1_ (275), *stx*_2_ (275), *stx*_2f_ (275), PhHv (200), O26 (370), O103 (320), O111 (320), and O145 (375). Raw *C_t_* values at these thresholds were exported in csv format for further data management and analysis within the R statistical system (R Core Team 2020) (82).

In order to standardize classification of samples to either a positive or negative status across all serogroups, we designated the *C_t_* cutoff value for each target serogroup or *stx* as that equivalent to the *C_t_* observed in the respective positive-control dilution series at a bacterial concentration equivalent to 1 × 10^4^ CFU/ml postenrichment. This was the equivalent minimum concentration reliably observed within the spiked dilution series extracts across all the different serogroup and *stx* (PP+) control strains. This cutoff value was calculated on an individual, plate-specific basis, using linear regression of the serial dilution *C_t_* values and starting CFU/ml to impute the cutoff at 1 × 10^4^ CFU/ml. This cutoff *C_t_* value was applied to each test sample mean duplicate well result to allocate the sample status on a plate-specific basis as either positive or negative.

In addition, the mean 1 × 10^4^ CFU/ml *C_t_* cutoff value across all plates for a given target was calculated from the individual plate-specific cutoffs to obtain a mean global value. Global *C_t_* cutoffs were calculated for O26 (35.28), O103 (37.08), O111 (36.43), O145 (36.98), *stx*_1_ (35.41), and *stx*_2_ (34.63). The global cutoff was applied to all sample results to allocate status additionally at the global level. Both the plate-specific and global sample status results were used in subsequent prevalence estimate calculations. All samples that were designated as having a negative status across all test PCR targets had a confirmed positive signal for the internal control target PhHv.

A proportion of samples underwent repeat PCR either of the existing DNA extract (technical replicate), particularly where sample status determined overall herd status, or for the routine 20th sample repeat DNA extract (biological replicate) (Tables S1 and S2). Where replicate status did not agree, a sample was designated “Border” and the prevalence estimate statistical calculations were repeated, with sample status results read at both plate-specific and global cutoffs and Border results allocated to first negative status and then positive status (Fig. S1 and Tables S3 to S10). For all analyses reported in the results section for the study, the more conservative measure of global cutoff, with Border samples allocated to negative status, was used.

### Isolation and identification of O26 strains.

Strain isolation was attempted from a subset of samples in all herds testing O26 PCR positive, selecting those with the lowest recorded O26 *C_t_* values. A maximum of eight samples were attempted per herd, where available. We did not attempt strain isolation from every positive sample in a herd, due to practical and financial constraints. Fecal enrichments were thawed on ice, diluted 1:5 with BPW (Thermo Fisher Scientific, Loughborough, UK), and incubated overnight with shaking at 37°C and 190 rpm. Two methods were used to attempt O26 strain isolation: (i) direct culture, by plating 20 μl of overnight culture onto CHROMagar STEC agar (CHROMagar, Paris, France), and (ii) immunomagnetic separation (IMS) using the semiautomated KingFisher mL machine (Thermo Fisher Scientific). For IMS, 1 ml of overnight culture was mixed with 20 μl of Dynabeads EPEC/VTEC O26 (Applied Biosystems, Thermo Fisher Scientific) and allowed to bind for 25 min, washed three times in phosphate-buffered saline (PBS)-Tween 20 (Sigma-Aldrich, Gillingham, UK) for 3 min each, and then eluted in PBS-Tween 20. Eluted beads were plated in 20 μl volumes onto each of CHROMagar STEC agar and UTI ChromoSelect agar (Sigma-Aldrich). Inoculated plates were incubated overnight at 37°C. Up to 7 individual colonies were picked from all available plates, selecting mauve colonies on CHROMagar STEC agar and pink-red colonies from UTI ChromoSelect. Picks were subcultured overnight on LB agar at 37°C, and resulting pure cultures of putative strains were stored on cryogenic Microbank beads (Pro-Lab Diagnostics Inc., ON, Canada), frozen at −80°C.

Latex agglutination and real-time PCR were used to confirm whether isolated strains were of the O26 serogroup. Latex agglutination was performed using the Prolex E. coli non-O157 kit (Pro-Lab Diagnostics Inc.), following the manufacturer’s instructions. Real-time PCR was as described previously. Strain serogroup and *stx* subtype were further confirmed by whole-genome sequencing. Putative O26 strains were sequenced at the SERL using the Illumina MiSeq (Illumina, CA, U.S.) by paired-end sequencing (60). Sequence data were analyzed by the Public Health England bioinformatics pipeline installed at the SERL and by BioNumerics v7.6 (Applied Maths, Ghent, Belgium) using the wgMLST and E. coli genotyping plug-in tools (60).

### Statistical analysis.

Chi square and Mann-Whitney U tests were performed in Minitab version 18.1 (Minitab Inc., PA, U.S.). A five-way Venn diagram was generated using the online tool provided at http://bioinformatics.psb.ugent.be/webtools/Venn/ (Bioinformatics and Evolutionary Genomics, University of Ghent, Belgium). Tetrachoric correlation analysis was performed on binary data using polycor in R.

### Prevalence estimates.

Herd-level and pat-level prevalence were estimated using a method similar to that of Henry et al. (34). Briefly, a generalized linear mixed model with a logit link fitted with a random herd effect to model extrabinomial variability was performed using Proc Glimmix (SAS version 9.4). Mean estimates and CIs were generated by back transforming from the output on the logit scale. Overall prevalence was estimated for each serogroup (O103, O145, O26) and *stx* subtype (*stx*_1_, *stx*_2_). O111 was not included due to insufficient numbers. Prevalence estimates were also calculated for different spatial (animal health district [AHD]) and temporal (season) factors. To enable comparisons with previous survey results, Scotland was divided into six AHDs (Highlands, Islands, Central, North East, South East, and South West). Season was defined according to the following: spring (March to May), summer (June to August), autumn (September to November), and winter (December to February).

### Risk associations.

Associations between the farm questionnaire data and herd-level presence/absence of non-O157 serogroups, together with STEC O157 (34), were performed using nonmetric multidimensional scaling (NMS). PC-ORD software version 7.04 (MJM Software Design, Gleneden Beach, OR) was used and analysis was performed on sample status results read at the global cutoff, with Border samples allocated to negative status. NMS was used with a grower distance measure. The dimensionality of the data set was determined by plotting an inverse measure of fit (“stress”) to the number of dimensions. Optimal dimensionality was based on the number of dimensions with the lowest stress. A two-dimensional solution was shown to be optimal. Several NMS runs were performed for each analysis to ensure that the solution was stable and represented a configuration with the best possible fit. On this basis, 500 iterations were used for each NMS run, using random starting coordinates.

## Supplementary Material

Supplemental file 1
