# Supplementary material for: Prevalence and Epidemiology of Non-O157 Escherichia coli Serogroups O26, O103, O111, and O145 and Shiga Toxin Gene Carriage in Scottish Cattle, 2014–2015
Source: Appl Environ Microbiol. 2021 Apr 27;87(10):e03142-20. doi: 10.1128/AEM.03142-20 (PMC8117755; doi:10.1128/AEM.03142-20)
Supplement: Supplemental file 1 [file AEM.03142-20-s0001.pdf]

Supplemental Data

Biological and Technical Replicates

There were 133 faecal pat enrichment samples that underwent a repeat DNA extraction procedure as biological replicates (BR), followed by real-time PCR for all three reactions. Technical replicates (TR), where a repeat PCR was performed on the same DNA extract on a different plate, were available for differing sample numbers depending on the PCR assay. Agreement was high, with less than 6% of BR and 7% of TR samples showing differential status across the cut-off threshold (Tables S1, S2). Where either BR or TR replicate status did not agree a sample was designated "Border". In order to examine the sensitivity of the prevalence estimates to the different cut-off methods, as well as the impact of "Border" samples, the calculations were performed on the following combinations: (i) plate specific cut-off with "Borders" classed negative, (ii) plate specific cut-off with "Borders" classed positive (iii) global cut-off with "Borders" classed negative, and (iv) global cut-off with "Borders" classed positive. The results are presented in Tables S3-S10 and summarised in Figure S1.

The prevalence figures and correlation analyses presented in the main manuscript, and the data for the nonmetric multidimensional scaling ordination analysis, were based on the more conservative measure of global cut-off, with "Border" samples allocated to negative status.

Table S1: Agreement in PCR sample status for Biological Replicates (n = 133)

	Global cut-off				Plate-specific cut-off			
	Agree		Border		Agree		Border	
	n	%	n	%	n	%	n	%
**O26**	130	97.7	3	2.3	130	97.7	3	2.3
**O103**	133	100.0	0	0.0	131	98.5	2	1.5
**O111**	132	99.2	1	0.8	132	99.2	1	0.8
**O145**	133	100.0	0	0.0	132	99.2	1	0.8
***stx*1**	127	95.5	6	4.5	131	98.5	2	1.5
***stx*2**	131	98.5	2	1.5	126	94.7	7	5.3

Table S2: Agreement in PCR sample status for Technical Replicates (numbers of Technical Replicates performed for each assay denoted by TR n)

		Global cut-off				Plate-specific cut-off			
		Agree		Border		Agree		Border	
	TR n	n	%	n	%	n	%	n	%
**O26**	407	396	97.3	11	2.7	393	96.6	14	3.4
**O103**	407	390	95.8	17	4.2	391	96.1	16	3.9
**O111**	407	403	99.0	4	1.0	402	98.8	5	1.2
**O145**	215	206	95.8	9	4.2	201	93.5	14	6.5
***stx*1**	493	480	97.4	13	2.6	474	96.1	19	3.9
***stx*2**	493	461	93.5	32	6.5	469	95.1	24	4.9

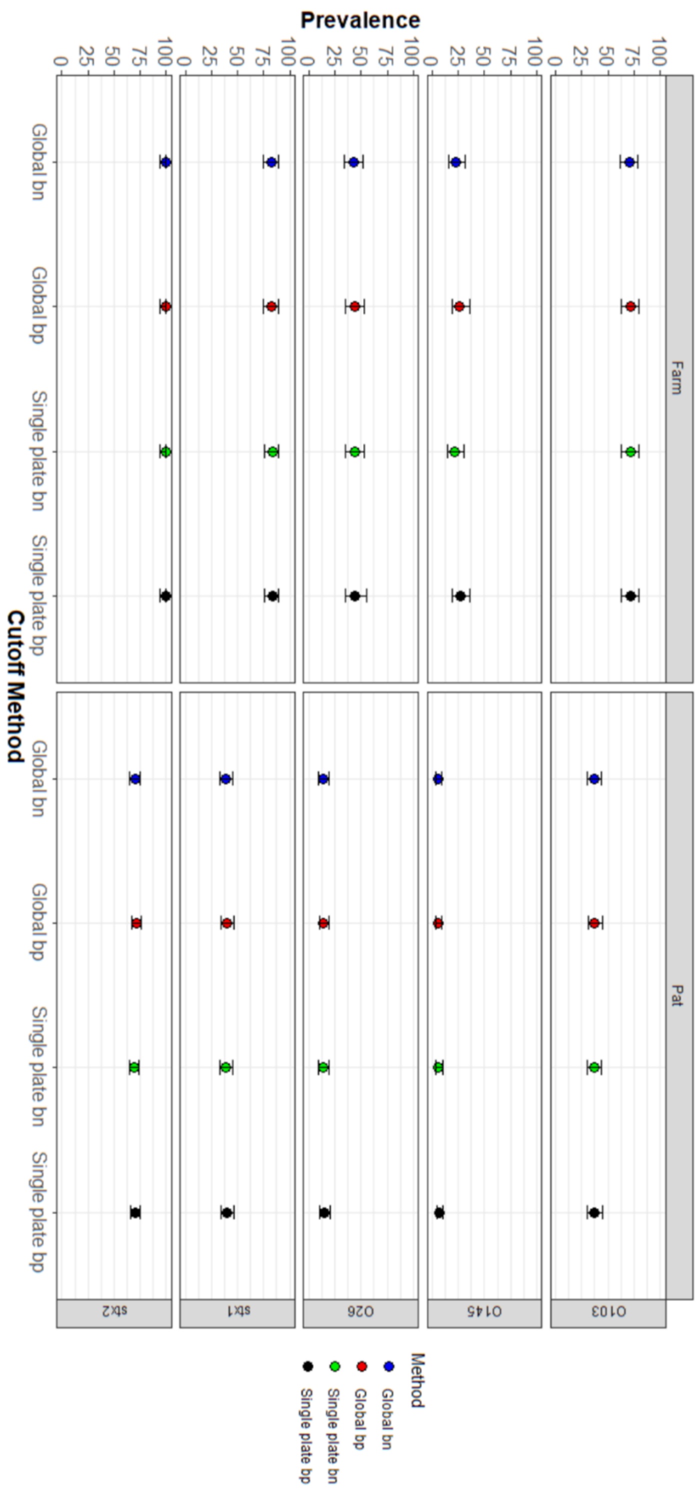

Fig. S1: Comparison of the overall herd (farm) and pat prevalence estimates for O26, O103, O145 serogroups, *stx*1 and *stx*2 genes by real-time PCR, at both the plate-specific (single plate) and global (mean plate) cut-off values, with “Border” samples set to negative (bn) and positive (bp); error bars denote 95% CI.

Table S3: Herd level prevalence estimates for sample results read at the PCR Global cut-off, with “Border” replicate sample results set to a “Negative” status, showing lower and upper 95% confidence intervals (CI)

Herd level; Global cut-off; "Border" samples set to "Negative" status						
		**Fixed effect**		**Prevalence estimate**		
**Analysis level**	**Variable**	**Estimate**	**SE**	**Mean**	**Lower CI**	**Upper CI**
**Serogroup/*stx***						
O26	intercept	-0.293	0.194	42.73	33.70	52.27
O103	intercept	0.891	0.211	70.91	61.61	78.73
O145	intercept	-1.224	0.229	22.73	15.75	31.63
*stx*1	intercept	1.566	0.253	82.73	74.35	88.78
*stx*2	intercept	4.691	1.009	99.09	93.65	99.88
**AHD: O26**	**DF=5,104; F=1.69; *P*=0.1442**					
CENTRAL	intercept	-1.099	0.591	25.00	9.36	51.83
HIGHLAND	intercept	-0.105	0.474	47.37	25.98	69.77
ISLANDS	intercept	-1.099	0.591	25.00	9.36	51.83
NE	intercept	-0.539	0.489	36.84	18.09	60.64
SE	intercept	-0.118	0.501	47.06	24.72	70.65
SW	intercept	0.629	0.450	65.22	43.43	82.08
**SEASON: O26**	**DF=3,106; F=1.96; *P*=0.1309**					
AUTUMN	intercept	0.357	0.355	58.82	41.38	74.30
WINTER	intercept	-0.511	0.430	37.50	20.38	58.45
SPRING	intercept	-0.916	0.425	28.57	14.69	48.17
SUMMER	intercept	-0.337	0.422	41.67	23.61	62.28
**AHD: O145**	**DF=5,104; F=0.35; *P*=0.8826**					
CENTRAL	intercept	-1.946	0.769	12.50	3.01	39.64
HIGHLAND	intercept	-0.773	0.511	31.58	14.29	56.09
ISLANDS	intercept	-1.099	0.595	25.00	9.28	52.06
NE	intercept	-1.322	0.578	21.05	7.82	45.60
SE	intercept	-1.179	0.588	23.53	8.74	49.71
SW	intercept	-1.281	0.519	21.74	9.03	43.75
**SEASON: O145**	**DF=3,106; F=4.67; *P*=0.0042**					
AUTUMN	intercept	-0.118	0.352	47.06	30.47	64.32
WINTER	intercept	-3.136	1.025	4.17	0.57	24.93
SPRING	intercept	-1.792	0.546	14.29	5.34	32.99
SUMMER	intercept	-1.609	0.555	16.67	6.24	37.54
**AHD: O103**	**DF=5,104; F=1.43; *P*=0.2179**					
CENTRAL	intercept	-2.7E-15	0.518	50.00	26.24	73.76
HIGHLAND	intercept	0.773	0.509	68.42	44.08	85.62
ISLANDS	intercept	0.511	0.534	62.50	36.54	82.83
NE	intercept	1.030	0.535	73.68	49.20	89.01
SE	intercept	0.876	0.548	70.59	44.71	87.69
SW	intercept	2.351	0.748	91.30	70.42	97.89
**SEASON: O103**	**DF=3,106; F=2.27; *P*=0.0850**					
AUTUMN	intercept	2.015	0.538	88.24	72.10	95.61
WINTER	intercept	0.337	0.424	58.33	37.57	76.51
SPRING	intercept	0.916	0.426	71.43	51.76	85.35

SUMMER	intercept	0.337	0.424	58.33	37.57	76.51
**AHD: *stx*1**	**DF=5,104; F=1.07; *P*=0.3809**					
CENTRAL	intercept	0.789	0.561	68.75	41.63	87.16
HIGHLAND	intercept	2.140	0.761	89.47	65.28	97.46
ISLANDS	intercept	1.466	0.659	81.25	53.96	94.13
NE	intercept	1.030	0.540	73.68	48.75	89.18
SE	intercept	1.540	0.653	82.35	56.09	94.46
SW	intercept	3.091	1.031	95.65	74.03	99.41
**SEASON: *stx*1**	**DF=3,106; F=2.54; *P*=0.0607**					
AUTUMN	intercept	2.015	0.539	88.24	72.05	95.62
WINTER	intercept	0.693	0.444	66.67	45.01	83.01
SPRING	intercept	3.296	1.023	96.43	78.05	99.51
SUMMER	intercept	1.099	0.482	75.00	53.41	88.70

Table S4: Herd level prevalence estimates for sample results read at the PCR Global cut-off, with “Border” replicate sample results set to a “Positive” status, showing lower and upper 95% confidence intervals (CI)

**Herd level; Global cut-off; "Border" samples set to "Positive" status**						
		**Fixed effect**		**Prevalence estimate**		
**Analysis level**	**Variable**	**Estimate**	**SE**	**Mean**	**Lower CI**	**Upper CI**
**Serogroup/*stx***						
O26	intercept	-0.256	0.193	43.64	34.55	53.17
O103	intercept	0.936	0.213	71.82	62.56	79.53
O145	intercept	-1.027	0.217	26.36	18.88	35.52
*stx*1	intercept	1.566	0.253	82.73	74.35	88.78
*stx*2	intercept	4.691	1.009	99.09	93.65	99.88
**AHD: O26**	**DF=5,104; F=1.78; *P*=0.1229**					
CENTRAL	intercept	-1.099	0.591	25.00	9.36	51.83
HIGHLAND	intercept	0.105	0.474	52.63	30.23	74.02
ISLANDS	intercept	-1.099	0.591	25.00	9.36	51.83
NE	intercept	-0.539	0.489	36.84	18.09	60.64
SE	intercept	-0.118	0.501	47.06	24.72	70.65
SW	intercept	0.629	0.450	65.22	43.43	82.08
**SEASON: O26**	**DF=3,106; F=2.33; *P*=0.0789**					
AUTUMN	intercept	0.480	0.360	61.76	44.18	76.73
WINTER	intercept	-0.511	0.430	37.50	20.37	58.45
SPRING	intercept	-0.916	0.425	28.57	14.69	48.17
SUMMER	intercept	-0.337	0.422	41.67	23.60	62.29
**AHD: O145**	**DF=5,104; F=0.07; *P*=0.9966**					
CENTRAL	intercept	-1.099	0.593	25.00	0.33	51.93
HIGHLAND	intercept	-0.773	0.509	31.58	14.48	55.92
ISLANDS	intercept	-1.099	0.593	25.00	0.33	51.93
NE	intercept	-1.030	0.536	26.32	10.99	50.81
SE	intercept	-1.179	0.587	23.53	8.77	49.61
SW	intercept	-1.042	0.488	26.09	11.82	48.16
**SEASON: O145**	**DF=3,106; F=3.90; *P*=0.0110**					
AUTUMN	intercept	-0.118	0.352	47.06	30.56	64.23
WINTER	intercept	-2.398	0.744	8.33	2.04	28.43
SPRING	intercept	-1.792	0.546	14.29	5.34	32.99
SUMMER	intercept	-0.887	0.458	29.17	14.22	50.56
**AHD: O103**	**DF=5,104; F=1.51; *P*=0.1928**					
CENTRAL	intercept	1.3E-15	0.518	50.00	26.21	73.79
HIGHLAND	intercept	0.773	0.509	68.42	44.05	85.64
ISLANDS	intercept	0.511	0.534	62.50	36.51	82.85
NE	intercept	1.030	0.536	73.68	49.18	89.01
SE	intercept	1.179	0.587	76.47	50.38	91.23
SW	intercept	2.351	0.785	91.30	70.41	97.89
**SEASON: O103**	**DF=3,106; F=2.27; *P*=0.0850**					
AUTUMN	intercept	2.015	0.538	88.24	72.09	95.61
WINTER	intercept	0.337	0.424	58.33	37.56	76.52
SPRING	intercept	0.916	0.426	71.43	51.75	85.35

SUMMER	intercept	0.511	0.431	62.50	41.39	79.73
**AHD: *stx*1**	**DF=5,104; F=1.07; *P*=0.3809**					
CENTRAL	intercept	0.789	0.561	68.75	41.63	87.16
HIGHLAND	intercept	2.140	0.761	89.47	65.28	97.46
ISLANDS	intercept	1.466	0.659	81.25	53.96	94.13
NE	intercept	1.030	0.540	73.68	48.75	89.18
SE	intercept	1.540	0.653	82.35	56.09	94.46
SW	intercept	3.091	1.031	95.65	74.03	99.41
**SEASON: *stx*1**	**DF=3,106; F=2.54; *P*=0.0607**					
AUTUMN	intercept	2.015	0.539	88.24	72.05	95.62
WINTER	intercept	0.693	0.444	66.67	45.01	83.01
SPRING	intercept	3.296	1.023	96.43	78.05	99.51
SUMMER	intercept	1.099	0.482	75.00	53.41	88.70

Table S5: Herd level prevalence estimates for sample results read at the PCR Plate-specific cut-off, with "Border" replicate sample results set to a "Negative" status, showing lower and upper 95% confidence intervals (CI)

**Herd level; Plate-specific cut-off; "Border" samples set to "Negative" status**						
		**Fixed effect**		**Prevalence estimate**		
**Analysis level**	**Variable**	**Estimate**	**SE**	**Mean**	**Lower CI**	**Upper CI**
**Serogroup/*stx***						
O26	intercept	-0.256	0.193	43.64	34.55	53.17
O103	intercept	0.954	0.213	71.82	62.56	79.53
O145	intercept	-1.276	0.232	21.82	14.98	30.65
*stx*1	intercept	1.631	0.259	83.64	75.37	89.52
*stx*2	intercept	4.691	1.009	99.09	93.65	99.88
**AHD: O26**	**DF=5,104; F=1.78; *P*=0.1229**					
CENTRAL	intercept	-1.099	0.591	25.00	9.361	51.83
HIGHLAND	intercept	0.105	0.474	52.63	30.23	74.02
ISLANDS	intercept	-1.099	0.591	25.00	9.36	51.83
NE	intercept	-0.539	0.489	36.84	18.09	60.64
SE	intercept	-0.118	0.501	47.06	24.72	70.65
SW	intercept	0.629	0.450	65.22	43.43	82.08
**SEASON: O26**	**DF=3,106; F=2.33; *P*=0.0789**					
AUTUMN	intercept	0.480	0.360	61.76	44.18	76.73
WINTER	intercept	-0.511	0.430	37.50	20.37	58.45
SPRING	intercept	-0.916	0.425	28.57	14.69	48.17
SUMMER	intercept	-0.337	0.422	41.67	23.60	62.29
**AHD: O145**	**DF=5,104; F=0.58; *P*=0.7161**					
CENTRAL	intercept	-2.708	1.042	6.25	0.84	34.50
HIGHLAND	intercept	-0.773	0.510	31.58	14.28	56.11
ISLANDS	intercept	-1.099	0.594	25.00	9.28	52.07
NE	intercept	-1.322	0.578	21.05	7.82	45.60
SE	intercept	-1.179	0.588	23.53	8.74	49.72
SW	intercept	-1.281	0.519	21.74	9.03	43.74
**SEASON: O145**	**DF=3,106; F=5.05; *P*=0.0026**					
AUTUMN	intercept	-0.118	0.352	47.06	30.44	64.35
WINTER	intercept	-3.136	1.025	4.17	0.57	24.93
SPRING	intercept	-1.792	0.546	14.29	5.34	32.99
SUMMER	intercept	-1.946	0.624	12.50	3.98	32.97
**AHD: O103**	**DF=5,104; F=1.51; *P*=0.1928**					
CENTRAL	intercept	-1.3E-15	0.518	50.00	26.21	73.79
HIGHLAND	intercept	0.773	0.509	68.42	44.05	85.64
ISLANDS	intercept	0.511	0.534	62.50	36.51	82.85
NE	intercept	1.030	0.536	73.68	49.18	89.01
SE	intercept	1.179	0.587	76.47	50.38	91.23
SW	intercept	2.351	0.749	91.30	70.41	97.89
**SEASON: O103**	**DF=3,106; F=2.27; *P*=0.0850**					
AUTUMN	intercept	2.015	0.538	88.24	72.09	95.61
WINTER	intercept	0.337	0.424	58.33	37.56	76.52
SPRING	intercept	0.916	0.426	71.43	51.75	85.35

SUMMER	intercept	0.511	0.431	62.50	41.39	79.73
**AHD: *stx*1**	**DF=5,104; F=1.16; *P*=0.3359**					
CENTRAL	intercept	0.789	0.561	68.75	41.54	87.20
HIGHLAND	intercept	2.140	0.761	89.47	65.27	97.47
ISLANDS	intercept	1.946	0.772	87.50	60.24	97.00
NE	intercept	1.030	0.540	73.68	48.68	89.21
SE	intercept	1.540	0.654	82.35	56.03	94.47
SW	intercept	3.091	1.031	95.65	74.02	99.41
**SEASON: *stx*1**	**DF=3,106; F=2.43; *P*=0.0693**					
AUTUMN	intercept	2.015	0.539	88.24	72.03	95.62
WINTER	intercept	0.693	0.445	66.67	44.94	83.05
SPRING	intercept	3.296	1.023	96.43	78.05	99.51
SUMMER	intercept	1.335	0.513	79.17	57.79	91.34

Table S6: Herd level prevalence estimates for sample results read at the PCR Plate-specific cut-off, with "Border" replicate sample results set to a "Positive" status, showing lower and upper 95% confidence intervals (CI)

**Herd level; Plate-specific cut-off; "Border" samples set to "Positive" status**						
		**Fixed effect**		**Prevalence estimate**		
**Analysis level**	**Variable**	**Estimate**	**SE**	**Mean**	**Lower CI**	**Upper CI**
**Serogroup/*stx***						
O26	intercept	-0.256	0.193	43.64	34.55	55.17
O103	intercept	0.954	0.213	71.82	62.56	79.53
O145	intercept	-0.981	0.215	27.27	19.67	36.48
*stx*1	intercept	1.631	0.259	83.64	75.37	89.52
*stx*2	intercept	4.691	1.009	99.09	93.65	99.88
**AHD: O26**	**DF=5,104; F=1.78; *P*=0.1229**					
CENTRAL	intercept	-1.099	0.591	25.00	9.36	51.83
HIGHLAND	intercept	0.105	0.474	52.63	30.23	74.02
ISLANDS	intercept	-1.099	0.591	25.00	9.36	51.83
NE	intercept	-0.539	0.489	36.84	18.09	60.64
SE	intercept	-0.118	0.501	47.06	24.72	70.65
SW	intercept	0.629	0.450	65.22	43.43	82.08
**SEASON: O26**	**DF=3,106; F=2.33; *P*=0.0789**					
AUTUMN	intercept	0.480	0.360	61.76	44.18	76.73
WINTER	intercept	-0.511	0.430	37.50	20.37	58.45
SPRING	intercept	-0.916	0.425	28.57	14.69	48.17
SUMMER	intercept	-0.337	0.422	41.67	23.60	62.29
**AHD: O145**	**DF=5,104; F=0.09; *P*=0.9932**					
CENTRAL	intercept	-1.099	0.593	25.00	0.33	51.93
HIGHLAND	intercept	-0.773	0.509	31.58	14.48	55.92
ISLANDS	intercept	-0.789	0.556	31.25	13.09	57.84
NE	intercept	-1.030	0.536	26.32	10.99	50.81
SE	intercept	-1.179	0.587	23.53	8.77	49.61
SW	intercept	-1.042	0.488	26.09	11.82	48.16
**SEASON: O145**	**DF=3,106; F=3.48; *P*=0.0185**					
AUTUMN	intercept	-0.118	0.352	47.06	30.56	64.23
WINTER	intercept	-1.946	0.624	12.50	3.99	32.96
SPRING	intercept	-1.792	0.546	14.29	5.34	32.99
SUMMER	intercept	-0.887	0.458	29.17	14.22	50.56
**AHD: O103**	**DF=5,104; F=1.51; *P*=0.1928**					
CENTRAL	intercept	1.3E-15	0.518	50.00	26.21	73.79
HIGHLAND	intercept	0.773	0.509	68.42	44.05	85.64
ISLANDS	intercept	0.511	0.534	62.50	36.51	82.85
NE	intercept	1.030	0.536	73.68	49.18	89.01
SE	intercept	1.179	0.587	76.47	50.38	91.23
SW	intercept	2.351	0.785	91.30	70.41	97.89
**SEASON: O103**	**DF=3,106; F=2.27; *P*=0.0850**					
AUTUMN	intercept	2.015	0.538	88.24	72.09	95.61
WINTER	intercept	0.337	0.424	58.33	37.56	76.52
SPRING	intercept	0.916	0.426	71.43	51.75	85.35

SUMMER	intercept	0.511	0.431	62.50	41.39	79.73
**AHD: *stx*1**	**DF=5,104; F=1.16; *P*=0.3359**					
CENTRAL	intercept	0.789	0.561	68.75	41.54	87.20
HIGHLAND	intercept	2.140	0.761	89.47	65.27	97.47
ISLANDS	intercept	1.946	0.772	87.50	60.24	97.00
NE	intercept	1.030	0.540	73.68	48.68	89.21
SE	intercept	1.540	0.654	82.35	56.03	94.47
SW	intercept	3.091	1.031	95.65	74.02	99.41
**SEASON: *stx*1**	**DF=3,106; F=2.43; *P*=0.0693**					
AUTUMN	intercept	2.015	0.539	88.24	72.03	95.62
WINTER	intercept	0.693	0.445	66.67	44.94	83.05
SPRING	intercept	3.296	1.023	96.43	78.05	99.51
SUMMER	intercept	1.335	0.513	79.17	57.79	91.34

Table S7: Pat level prevalence estimates for sample results read at the PCR Global cut-off, with “Border” replicate sample results set to a “Negative” status, showing lower and upper 95% confidence intervals (CI)

Pat level; Global cut-off; "Border" samples set to "Negative" status						
		**Fixed effect**		**Prevalence estimate**		
**Analysis level**	**Variable**	**Estimate**	**SE**	**Mean**	**Lower CI**	**Upper CI**
**Serogroup/*stx***						
O26	intercept	-1.837	0.208	13.74	9.54	19.39
O103	intercept	-0.534	0.152	36.95	30.24	44.20
O145	intercept	-2.840	0.278	5.52	3.26	9.20
*stx*1	intercept	-0.446	0.132	39.04	33.03	45.40
*stx*2	intercept	0.842	0.114	69.88	64.91	74.42
**AHD: O26**	**DF=5,104; F=0.75; *P*=0.5850**					
CENTRAL	intercept	-2.793	0.611	5.77	1.79	17.06
HIGHLAND	intercept	-2.080	0.539	11.11	4.12	26.68
ISLANDS	intercept	-1.959	0.591	12.36	4.19	31.30
NE	intercept	-1.773	0.535	14.52	5.56	32.90
SE	intercept	-1.282	0.560	21.72	8.37	45.75
SW	intercept	-1.662	0.486	15.95	6.75	33.23
**SEASON: O26**	**DF=3,106; F=0.50; *P*=0.6844**					
AUTUMN	intercept	-1.679	0.385	15.72	8.00	28.58
WINTER	intercept	-1.807	0.463	14.10	6.15	29.14
SPRING	intercept	-2.308	0.435	9.05	4.03	19.06
SUMMER	intercept	-1.653	0.466	16.08	7.07	32.53
**AHD: O145**	**DF=5,104; F=1.31; *P*=0.2675**					
CENTRAL	intercept	-2.788	0.681	5.80	1.57	19.21
HIGHLAND	intercept	-2.160	0.607	10.34	3.34	27.82
ISLANDS	intercept	-3.267	0.715	3.67	0.91	13.59
NE	intercept	-4.528	0.754	1.07	0.24	4.60
SE	intercept	-2.718	0.659	6.19	1.75	19.62
SW	intercept	-2.797	0.566	5.75	1.94	15.80
**SEASON: O145**	**DF=3,104.7; F=1.03; *P*=0.3677**					
AUTUMN	intercept	-2.352	0.546	8.69	3.12	21.98
WINTER	intercept	-3.245	0.680	3.75	1.00	13.05
SPRING	intercept	-3.780	0.649	2.23	0.63	7.64
SUMMER	intercept	-2.672	0.668	6.46	1.80	20.64
**AHD: O103**	**DF=5,104; F=1.66; *P*=0.1499**					
CENTRAL	intercept	-1.360	0.418	20.42	10.07	37.02
HIGHLAND	intercept	-0.686	0.377	33.50	19.26	51.53
ISLANDS	intercept	-0.211	0.413	44.75	26.32	64.74
NE	intercept	-0.781	0.378	31.40	17.79	49.19
SE	intercept	-0.611	0.399	35.18	19.73	54.50
SW	intercept	0.077	0.342	51.93	35.40	68.05
**SEASON: O103**	**DF=3,106; F=1.66; *P*=0.1792**					
AUTUMN	intercept	-0.075	0.279	48.13	34.79	61.75
WINTER	intercept	-0.873	0.338	29.47	17.63	44.93
SPRING	intercept	-0.549	0.309	36.61	23.82	51.61

SUMMER	intercept	-0.920	0.341	28.49	16.84	43.94
**AHD: *stx*2**	**DF=5,102.6; F=2.99; *P*=0.0146**					
CENTRAL	intercept	0.697	0.296	66.75	52.76	78.30
HIGHLAND	intercept	0.591	0.269	64.35	51.42	75.49
ISLANDS	intercept	0.252	0.297	56.27	41.65	69.87
NE	intercept	0.668	0.270	66.11	53.30	76.93
SE	intercept	1.402	0.296	80.25	69.32	87.97
SW	intercept	1.456	0.251	81.09	72.25	87.59
**SEASON: *stx*2**	**DF=3,103.9; F=0.91; *P*=0.4407**					
AUTUMN	intercept	0.651	0.203	65.72	56.18	74.15
WINTER	intercept	0.728	0.246	67.43	55.99	77.12
SPRING	intercept	0.957	0.227	72.26	62.41	80.34
SUMMER	intercept	1.136	0.252	75.70	65.39	83.71
**AHD: *stx*1**	**DF=5,103.9; F=0.86; *P*=0.5094**					
CENTRAL	intercept	-0.579	0.353	35.91	21.76	53.03
HIGHLAND	intercept	-0.260	0.322	43.55	28.94	59.37
ISLANDS	intercept	-0.906	0.360	28.78	16.53	45.19
NE	intercept	-0.729	0.325	32.54	20.23	47.86
SE	intercept	-0.288	0.342	42.86	27.57	59.64
SW	intercept	-0.122	0.293	46.96	33.10	61.29
**SEASON: *stx*1**	**DF=3, 104.6; F=1.84; *P*=0.1451**					
AUTUMN	intercept	-0.588	0.241	35.71	25.62	47.24
WINTER	intercept	-0.922	0.292	28.45	18.23	41.51
SPRING	intercept	-0.054	0.266	48.65	35.88	61.60
SUMMER	intercept	-0.279	0.292	43.08	29.79	57.45

Table S8: Pat level prevalence estimates for sample results read at the PCR Global cut-off, with “Border” replicate sample results set to a “Positive” status, showing lower and upper 95% confidence intervals (CI)

**Pat level; Global cut-off; "Border" samples set to "Positive" status**						
		**Fixed effect**		**Prevalence estimate**		
**Analysis level**	**Variable**	**Estimate**	**SE**	**Mean**	**Lower CI**	**Upper CI**
**Serogroup/*stx***						
O26	intercept	-1.803	0.206	14.15	9.88	19.87
O103	intercept	-0.510	0.151	37.52	30.81	44.75
O145	intercept	-2.779	0.263	5.85	3.55	9.49
*stx*1	intercept	-0.420	-0.132	39.66	33.60	46.06
*stx*2	intercept	0.906	0.114	71.22	66.39	75.61
**AHD: O26**	**DF=5,104; F=0.77; *P*=0.5764**					
CENTRAL	intercept	-2.755	0.606	5.98	1.88	17.47
HIGHLAND	intercept	-2.016	0.535	11.75	4.41	27.77
ISLANDS	intercept	-1.937	0.587	12.60	4.31	31.60
NE	intercept	-1.772	0.531	14.53	5.59	32.77
SE	intercept	-1.229	0.556	22.64	8.85	46.89
SW	intercept	-1.628	0.483	16.42	7.01	33.85
**SEASON: O26**	**DF=3,106; F=0.57; *P*=0.6364**					
AUTUMN	intercept	-1.640	0.381	16.25	8.35	29.24
WINTER	intercept	-1.747	0.459	14.84	6.56	30.21
SPRING	intercept	-2.307	0.431	9.05	4.06	18.97
SUMMER	intercept	-1.622	0.462	16.49	7.33	33.03
**AHD: O145**	**DF=5,104; F=1.18; *P*=0.3246**					
CENTRAL	intercept	-2.673	0.645	6.46	1.88	19.89
HIGHLAND	intercept	-2.140	0.577	10.53	3.61	27.01
ISLANDS	intercept	-3.159	0.678	4.07	1.10	14.00
NE	intercept	-4.236	0.692	1.43	0.37	5.40
SE	intercept	-2.712	0.628	6.23	1.87	18.75
SW	intercept	-2.744	0.538	6.04	2.16	15.76
**SEASON: O145**	**DF=3,104.3; F=1.27; *P*=0.2890**					
AUTUMN	intercept	-2.318	0.518	8.97	3.40	21.60
WINTER	intercept	-3.186	0.645	3.97	1.14	12.94
SPRING	intercept	-3.781	0.621	2.23	0.66	7.24
SUMMER	intercept	-2.535	0.631	7.35	2.21	21.73
**AHD: O103**	**DF=5,104; F=1.69; *P*=0.1439**					
CENTRAL	intercept	-1.351	0.414	20.57	10.22	37.06
HIGHLAND	intercept	-0.666	0.373	33.94	19.68	51.85
ISLANDS	intercept	-0.191	0.409	45.25	26.86	65.03
NE	intercept	-0.742	0.374	32.26	18.49	50.00
SE	intercept	-0.561	0.395	36.34	20.67	55.56
SW	intercept	0.092	0.339	52.29	35.87	68.22
**SEASON: O103**	**DF=3,106; F=1.66; *P*=0.1797**					
AUTUMN	intercept	-0.047	0.276	48.81	35.55	62.25
WINTER	intercept	-0.848	0.334	29.99	18.10	45.38
SPRING	intercept	-0.543	0.306	36.75	24.05	51.60

SUMMER	intercept	-0.872	0.338	29.48	17.64	44.94
**AHD: *stx*2**	**DF=5,102.6; F=2.99; *P*=0.0146**					
CENTRAL	intercept	0.697	0.294	66.75	52.85	78.24
HIGHLAND	intercept	0.756	0.269	68.05	55.55	78.40
ISLANDS	intercept	0.288	0.296	57.14	42.59	70.55
NE	intercept	0.731	0.269	67.50	54.91	77.98
SE	intercept	1.468	0.296	81.28	70.72	88.65
SW	intercept	1.508	0.251	81.88	73.32	88.14
**SEASON: *stx*2**	DF=3,103.6; F=1.29; *P*=0.2812					
AUTUMN	intercept	0.676	0.201	66.28	56.89	74.54
WINTER	intercept	0.766	0.243	68.27	57.05	77.70
SPRING	intercept	1.099	0.226	75.00	65.72	82.44
SUMMER	intercept	1.213	0.251	77.09	67.19	84.68
**AHD: *stx*1**	DF=5,104; F=0.93; *P*=0.4665					
CENTRAL	intercept	-0.573	0.354	36.05	21.83	53.22
HIGHLAND	intercept	-0.222	0.323	44.48	29.70	60.31
ISLANDS	intercept	-0.880	0.360	29.31	16.87	45.86
NE	intercept	-0.723	0.325	32.67	20.30	48.04
SE	intercept	-0.257	0.343	43.62	28.17	60.42
SW	intercept	-0.079	0.294	48.02	34.02	62.33
**SEASON: *stx*1**	DF=3,104.6; F=2.02; *P*=0.1152					
AUTUMN	intercept	-0.583	0.241	35.84	25.71	47.40
WINTER	intercept	-0.911	0.292	28.69	18.39	41.80
SPRING	intercept	-0.008	0.266	49.79	36.92	62.69
SUMMER	intercept	-0.233	0.292	44.19	30.73	58.57

Table S9: Pat level prevalence estimates for sample results read at the PCR Plate-specific cut-off, with "Border" replicate sample results set to a "Negative" status, showing lower and upper 95% confidence intervals (CI)

Pat level; Plate-specific cut-off; "Border" samples set to "Negative" status						
		**Fixed effect**		**Prevalence estimate**		
**Analysis level**	**Variable**	**Estimate**	**SE**	**Mean**	**Lower CI**	**Upper CI**
**Serogroup/*stx***						
O26	intercept	-1.824	0.208	13.90	9.65	19.61
O103	intercept	-0.536	0.153	36.90	30.15	44.21
O145	intercept	-2.754	0.267	5.99	3.62	9.76
*stx*1	intercept	-0.447	0.131	39.02	33.06	45.32
*stx*2	intercept	0.820	0.114	69.43	64.43	74.00
**AHD: O26**	**DF=5,104; F=0.76; *P*=0.5787**					
CENTRAL	intercept	-2.756	0.612	5.98	1.86	17.61
HIGHLAND	intercept	-2.083	0.541	11.08	4.09	26.70
ISLANDS	intercept	-1.976	0.594	12.18	4.10	31.04
NE	intercept	-1.768	0.537	14.57	5.56	33.09
SE	intercept	-1.230	0.562	22.62	8.75	47.14
SW	intercept	-1.654	0.488	16.05	6.78	33.48
**SEASON: O26**	**DF=3,106; F=0.68; *P*=0.5638**					
AUTUMN	intercept	-1.670	0.386	15.85	8.05	28.82
WINTER	intercept	-1.776	0.465	14.48	6.32	29.85
SPRING	intercept	-2.384	0.438	8.44	3.73	18.00
SUMMER	intercept	-1.586	0.467	16.99	7.51	34.04
**AHD: O145**	**DF=5,104; F=0.94; *P*=0.4601**					
CENTRAL	intercept	-2.827	0.698	5.59	1.46	19.13
HIGHLAND	intercept	-2.070	0.620	11.21	3.55	30.21
ISLANDS	intercept	-2.932	0.711	5.06	1.28	17.92
NE	intercept	-4.099	0.717	1.63	0.40	6.44
SE	intercept	-2.730	0.675	6.12	1.68	19.93
SW	intercept	-2.755	0.579	5.98	1.98	16.72
**SEASON: O145**	**DF=3,102.9; F=1.00; *P*=0.3965**					
AUTUMN	intercept	-2.193	0.550	10.04	3.61	24.96
WINTER	intercept	-3.245	0.686	3.75	0.99	13.18
SPRING	intercept	-3.560	0.643	2.77	0.79	9.24
SUMMER	intercept	-2.752	0.676	6.00	1.64	19.61
**AHD: O103**	**DF=5,104; F=1.68; *P*=0.1452**					
CENTRAL	intercept	-1.423	0.422	19.43	9.46	35.74
HIGHLAND	intercept	-0.716	0.379	32.82	18.72	50.90
ISLANDS	intercept	-0.217	0.416	44.60	26.10	64.73
NE	intercept	-0.732	0.380	32.47	18.46	50.53
SE	intercept	-0.560	0.402	36.36	20.48	55.89
SW	intercept	0.053	0.344	51.33	34.75	67.62
**SEASON: O103**	**DF=3,106; F=1.69; *P*=0.1746**					
AUTUMN	intercept	-0.076	0.281	48.11	34.67	61.83
WINTER	intercept	-0.916	0.341	28.57	16.92	44.00
SPRING	intercept	-0.535	0.312	36.93	23.99	52.08

SUMMER	intercept	-0.902	0.344	28.86	17.02	44.50
**AHD: *stx*2**	**DF=5,103.4; F=3.21; *P*=0.0098**					
CENTRAL	intercept	0.521	0.292	62.75	48.58	75.02
HIGHLAND	intercept	0.582	0.267	64.15	51.33	75.22
ISLANDS	intercept	0.243	0.294	56.04	41.57	69.56
NE	intercept	0.727	0.268	67.41	54.86	77.87
SE	intercept	1.394	0.293	80.12	69.25	87.82
SW	intercept	1.453	0.249	81.05	72.30	87.51
**SEASON: *stx*2**	**DF=3,104.4; F=0.59; *P*=0.6213**					
AUTUMN	intercept	0.775	0.204	68.47	59.17	76.49
WINTER	intercept	0.592	0.245	64.38	52.64	74.61
SPRING	intercept	0.913	0.227	71.36	61.36	79.63
SUMMER	intercept	1.030	0.252	73.69	62.98	82.18
**AHD: *stx*1**	**DF=5,103.9; F=0.80; *P*=0.5522**					
CENTRAL	intercept	-0.622	0.350	34.93	21.14	51.8
HIGHLAND	intercept	-0.303	0.319	42.47	28.17	58.16
ISLANDS	intercept	-0.887	0.356	29.18	16.90	45.50
NE	intercept	-0.683	0.321	33.56	21.08	48.84
SE	intercept	-0.242	0.339	43.97	28.63	60.57
SW	intercept	-0.140	0.290	46.50	32.82	60.72
**SEASON: *stx*1**	**DF=3,104.1; F=2.10; *P*=0.1048**					
AUTUMN	intercept	-0.568	0.240	36.17	26.05	47.68
WINTER	intercept	-0.996	0.291	26.97	17.17	39.67
SPRING	intercept	-0.098	0.264	47.55	34.93	60.49
SUMMER	intercept	-0.196	0.290	45.11	31.61	59.37

Table S10: Pat level prevalence estimates for sample results read at the PCR Plate-specific cut-off, with “Border” replicate sample results set to a “Negative” status, showing lower and upper 95% confidence intervals (CI)

Pat level; Plate-specific cut-off; "Border" samples set to "Positive" status						
		**Fixed effect**		**Prevalence estimate**		
**Analysis level**	**Variable**	**Estimate**	**SE**	**Mean**	**Lower CI**	**Upper CI**
**Serogroup/*stx***						
O26	intercept	-1.785	0.205	14.37	10.06	20.13
O103	intercept	-0.514	0.153	37.43	30.66	44.73
O145	intercept	-2.666	0.252	6.50	4.05	10.28
*stx*1	intercept	-0.415	0.113	39.77	33.74	46.12
*stx*2	intercept	0.874	0.114	70.56	65.66	75.03
**AHD: O26**	**DF=5,104; F=0.77; *P*=0.5714**					
CENTRAL	intercept	-2.719	0.601	6.19	1.95	17.99
HIGHLAND	intercept	-2.038	0.536	11.53	4.30	27.40
ISLANDS	intercept	-1.954	0.589	12.42	4.22	31.32
NE	intercept	-1.774	0.532	15.22	5.88	34.02
SE	intercept	-1.207	0.558	23.02	9.00	47.48
SW	intercept	-1.601	0.484	16.79	7.18	34.49
**SEASON: O26**	**DF=3,106; F=0.76; *P*=0.5166**					
AUTUMN	intercept	-1.631	0.381	16.37	8.42	29.43
WINTER	intercept	-1.735	0.459	14.99	6.63	30.45
SPRING	intercept	-2.370	0.433	8.55	3.81	18.06
SUMMER	intercept	-1.531	0.460	17.79	7.99	35.03
**AHD: O145**	**DF=5,104; F=0.91; *P*=0.4746**					
CENTRAL	intercept	-2.673	0.643	6.46	1.89	19.82
HIGHLAND	intercept	-2.054	0.573	11.37	3.94	28.60
ISLANDS	intercept	-2.816	0.657	5.65	1.60	18.05
NE	intercept	-3.886	0.656	2.01	0.56	7.01
SE	intercept	-2.697	0.626	6.32	1.91	18.91
SW	intercept	-2.613	0.533	6.83	2.48	17.45
**SEASON: O145**	**DF=3,102.9; F=1.19; *P*=0.3158**					
AUTUMN	intercept	-2.135	0.506	10.58	4.15	24.41
WINTER	intercept	-3.121	0.631	4.23	1.25	13.37
SPRING	intercept	-3.526	0.597	2.86	0.89	8.76
SUMMER	intercept	-2.583	0.621	7.03	2.16	20.58
**AHD: O103**	**DF=5,104; F=1.76; *P*=0.1287**					
CENTRAL	intercept	-1.422	0.420	19.43	9.49	35.66
HIGHLAND	intercept	-0.678	0.377	33.67	19.36	51.76
ISLANDS	intercept	-0.178	0.414	45.56	26.93	65.52
NE	intercept	-0.728	0.378	32.57	18.58	50.56
SE	intercept	-0.529	0.400	37.08	21.06	56.56
SW	intercept	-0.074	0.343	51.86	35.30	68.01
**SEASON: O103**	**DF=3,106; F=1.74; *P*=0.1635**					
AUTUMN	intercept	-0.044	0.280	48.89	35.46	62.49
WINTER	intercept	-0.910	0.339	28.70	17.06	44.06
SPRING	intercept	-0.523	0.310	37.22	24.28	52.29

SUMMER	intercept	-0.861	0.342	29.71	17.67	45.41
**AHD: *stx*2**	**DF=5,103.2; F=3.02; *P*=0.0138**					
CENTRAL	intercept	0.534	0.291	63.04	48.90	75.25
HIGHLAND	intercept	0.689	0.267	66.58	53.98	77.19
ISLANDS	intercept	0.320	0.294	57.92	43.43	71.17
NE	intercept	0.781	0.268	68.59	56.19	78.81
SE	intercept	1.046	0.293	80.31	69.50	87.95
SW	intercept	1.504	0.250	81.82	73.29	88.07
**SEASON: *stx*2**	**DF=3,104.1; F=0.72; *P*=0.5398**					
AUTUMN	intercept	0.792	0.203	68.82	59.59	76.76
WINTER	intercept	0.645	0.245	65.59	54.00	75.59
SPRING	intercept	1.013	0.227	73.35	63.70	81.19
SUMMER	intercept	1.093	0.251	74.90	64.45	83.08
**AHD: *stx*1**	**DF=5,103.9; F=0.84; *P*=0.5276**					
CENTRAL	intercept	-0.596	0.352	35.53	21.53	52.53
HIGHLAND	intercept	-0.261	0.320	43.50	28.97	59.24
ISLANDS	intercept	-0.857	0.357	28.90	17.29	46.31
NE	intercept	-0.665	0.322	33.95	21.34	49.35
SE	intercept	-0.229	0.340	44.30	28.83	60.95
SW	intercept	-0.088	0.292	47.79	33.92	62.01
**SEASON: *stx*1**	**DF=3,104.2; F=2.22; *P*=0.0904**					
AUTUMN	intercept	-0.540	0.240	36.81	26.56	48.42
WINTER	intercept	-0.979	0.292	27.31	17.39	40.14
SPRING	intercept	-0.039	0.265	49.03	36.24	61.94
SUMMER	intercept	-0.179	0.291	45.54	31.94	59.84
